# ProCV: A 3D similarity grouping method for enhanced protein pocket recognition and ligand interaction analysis

**DOI:** 10.1016/j.isci.2025.112305

**Published:** 2025-03-27

**Authors:** Zhenhao Wang, Tingyuan Nie

**Affiliations:** 1 School of Information and Control Engineering, Qingdao University of Technology, No.777 Jialingjiang East Road, West Coast New Area, Qingdao 266520, China; 2 Department of Electrical Engineering and Computer Science, Bond Life Sciences Center, University of Missouri, Columbia, MO 65211-7310, USA

**Keywords:** Biocomputational method, Software program for structure determination

## Abstract

Efficient identification of protein binding pockets is critical for accurately predicting protein-ligand interactions. Traditional sequence-based methods often fail to capture structural complexity and require extensive conformational sampling, limiting both efficiency and accuracy. To overcome these challenges, we present ProCV, an innovative structure-based prediction method that utilizes advanced spatial recognition techniques—specifically, 3D similarity grouping in the Hough space—to enhance precision and speed. ProCV employs uniform spatial sampling, KD-tree structures, and the 3D Hough transform for accurate binding pocket identification. Comparative analyses on datasets from the Protein DataBank (PDB), scPDB, and BioLip demonstrate that ProCV offers high specificity and sensitivity with reduced false positives. Its similarity assessment framework accurately characterizes the spatial arrangement of 3D protein structures, facilitating precise binding site localization. These findings highlight ProCV’s robustness, precision, and flexibility in identifying binding residues at atomic resolution within 3D structures, affirming its value in structural bioinformatics for protein-ligand interaction studies.

## Highlights

• Developed ProCV, a protein pocket recognition method using 3D similarity grouping
• Used KD-trees, spatial sampling, and 3D Hough transform for faster binding site detection
• Achieved superior pocket overlap score (POS) with strong noise resistance in matching
• Experimental results show ProCV’s effectiveness in structural bioinformatics studies

## Graphical abstract

**Figure:**
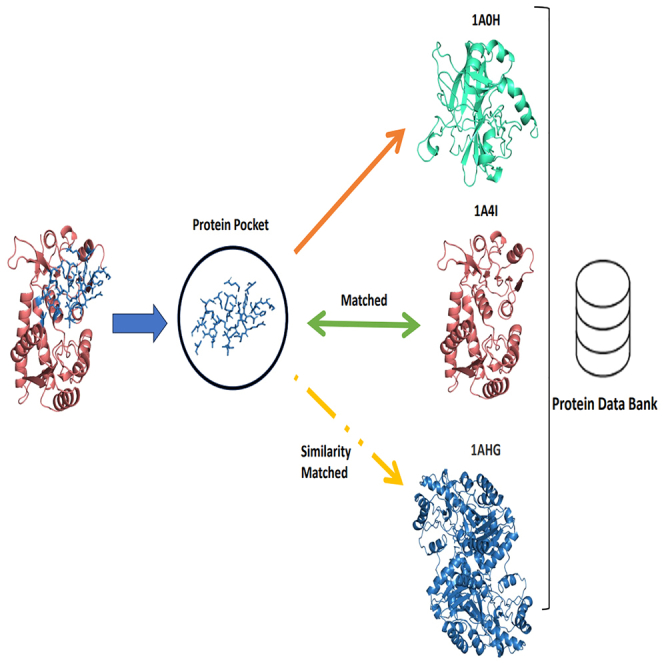

## Introduction

Proteins interact with small molecules through binding sites, which are essential for their function and druggability.^1^ Identifying these pockets is crucial for drug discovery, structural biology, and enzyme engineering. Traditional computational methods, such as PASS,^2^ LIGSITE,^3^ and Q-SiteFinder,^4^ detect binding pockets by analyzing protein surfaces, buried volumes, and van der Waals interactions using geometric and energy-based approaches.

Recent advances in machine learning and deep learning have significantly improved pocket detection. Graph neural network-based methods like PocketMiner^5^ efficiently predict cryptic pockets, while generative models like PocketGen^6^ co-design pocket structures and residue sequences, surpassing traditional physics-based techniques. Additionally, descriptor-based approaches such as PocketVec^7^ facilitate large-scale functional site comparisons, supporting drug repurposing efforts.

Integrating geometric, energy-based, and AI-driven approaches has enhanced protein binding site identification. Hybrid methods, such as blind docking combined with pocket search (BD + PS),^8^ refine functional pocket predictions, while probabilistic and sampling-based docking techniques like Q-Fit^9^ and SITEHOUND^10^ improve binding site rankings. These advancements contribute to structure-based drug discovery by enhancing accuracy, scalability, and efficiency.

A major milestone in structural bioinformatics is the development of AlphaFold, which has revolutionized protein structure prediction. By May 2024, the AlphaFold 3 database contained over 214 million structures, integrated into key resources such as the Protein DataBank (PDB), UniProt, and Ensembl.^11^^,^^12^^,^^13^^,^^14^ While sequence-based methods provide computational efficiency, structural data improves precision, offering deeper insights into protein dynamics and complex binding interactions.^15^

Emerging techniques leverage 3D point cloud representations of protein cavities annotated with pharmacophoric properties. Approaches using iterative closest point (ICP) alignment and fast point feature histograms (FPFH) enhance similarity detection in protein pockets, providing a rapid and accurate alternative to computationally intensive methods.^16^^,^^17^^,^^18^^,^^19^^,^^20^ Moreover, models like protein structure transformer (PeSTo) apply geometric transformations to protein atom coordinates, improving structural alignment and computational efficiency without explicitly encoding physical and chemical properties.^21^

Addressing challenges such as the curse of dimensionality and local optima, the cross-analytical ProCV method employs 3D spatial data to identify protein pockets with atomic precision, surpassing sequence-only approaches. By capitalizing on structural data, ProCV enhances binding residue prediction accuracy, providing a robust framework for binding site recognition.

This study focuses on protein pocket identification using structural data. We employ 3D reconstruction techniques to model protein pockets, utilizing resources such as PDB,^22^^,^^23^^,^^24^ scPDB,^25^ BioLip,^26^^,^^27^^,^^28^ and real-world datasets.^29^^,^^30^^,^^31^^,^^32^^,^^33^ Through atomic-level orientation and surface shape analysis in 3D point cloud data (PCD), and by leveraging KD-tree structures and the 3D Hough transform we enhance protein pocket visualization and recognition.

The remainder of this paper is organized as follows: Approaches outlines the theoretical foundations of our method, results presents the experimental findings, conclusion discusses the conclusions and future research directions, and discussion provides broader implications and insights.

### Approaches

#### Overview

**Figure 1:**
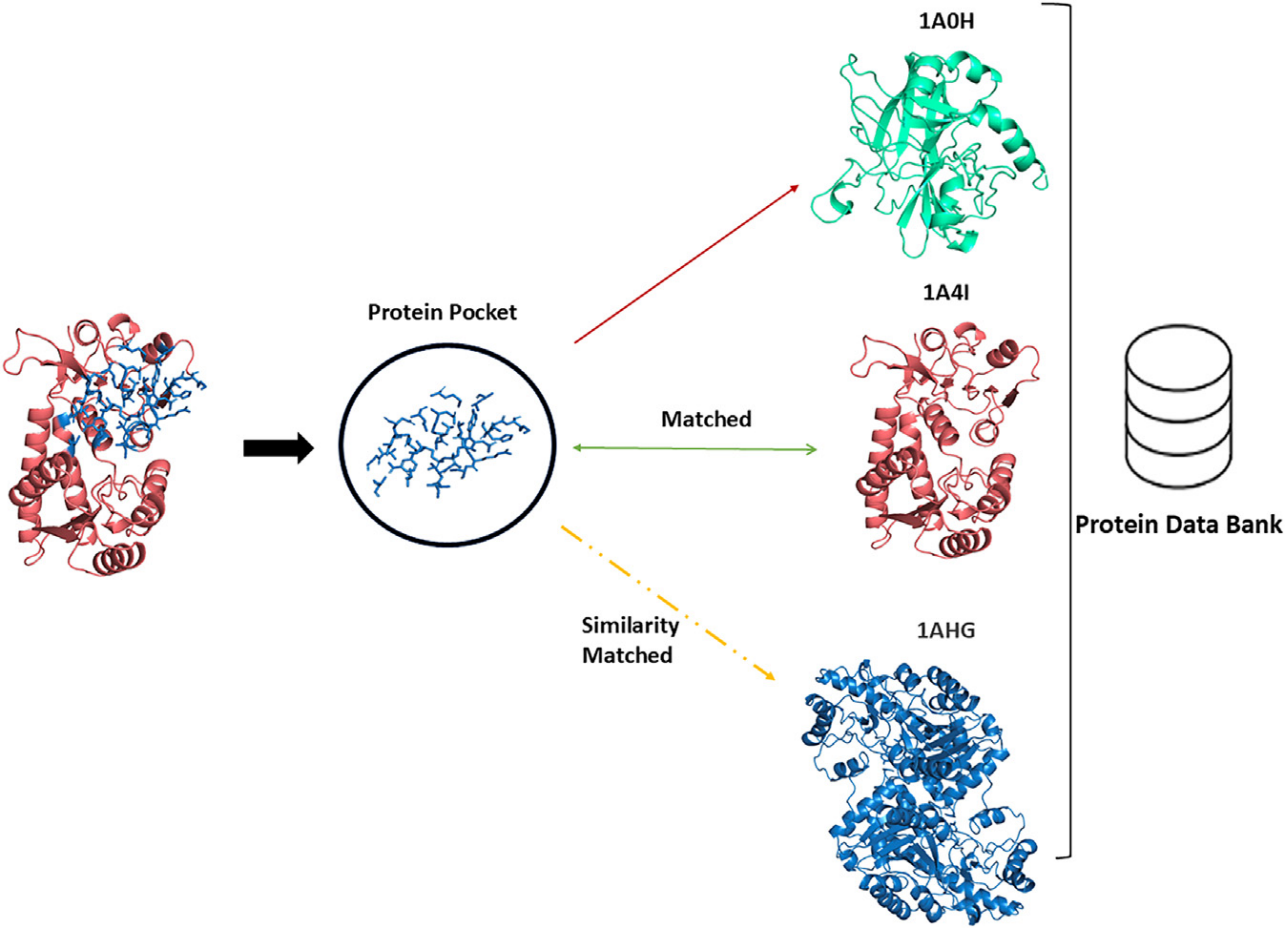
Schematic illustrating the matching and similarity estimation between proteins and a protein pocket in protein pocket recognition using ProCV

The diagram in Figure 1 presents a schematic illustrating the matching and similarity estimation between proteins and a protein pocket (binding site) in the context of protein pocket recognition using ProCV. These matches between protein pockets and a protein database are used to predict protein-ligand binding residues. On the left, the inputs are shown: the protein (1A4I) with its highlighted protein pocket (in blue). A close-up view of the protein pocket is provided, displaying the amino acid residues (in blue). The recognition of the protein pocket is defined as the area within a 5 Ångström (Å) radius around the pocket.

On the right, an example of matching the recognized protein pocket with structures from the Protein DataBank (PDB) database is shown. Three distinct protein structures from the PDB are displayed: 1A0H, 1A4I, and 1AHG, represented in green, red, and blue, respectively. The matching result, verified through a one-to-many comparison, demonstrates that the recognized protein pocket (in blue) was compared against the database to identify the exact matching protein structure (1A4I), with no errors, even in databases containing structural noise. Here, noise is defined as the positional deviation (δ) of atomic coordinates in the protein structure, calculated as:

(Equation 1) $$\delta =\sqrt{\frac{1}{N}\sum_{i=1}^{N}{\left(x_{i}^{\prime }-x_{i}\right)}^{2}+{\left(y_{i}^{\prime }-y_{i}\right)}^{2}+{\left(z_{i}^{\prime }-z_{i}\right)}^{2}}$$

where N is the total number of atoms, and $\left(x_{i}^{\prime },y_{i}^{\prime },z_{i}^{\prime }\right)$ and $\left(x_{i},y_{i},z_{i}\right)$ represent the perturbed and original atomic coordinates, respectively. Noise levels ranged from 0 to 1.5 Å in this study.

Additionally, the verification results display the similarity matching between the 1A4I pocket and protein 1AHG. This well-established similarity matching plays a crucial role in protein pocket recognition, particularly in drug discovery and structural biology.

The Protein DataBank (PDB) is a critical repository for three-dimensional (3D) structural data of biological macromolecules, including proteins, nucleic acids, and complex assemblies. The structural data in the PDB is derived from experimental techniques such as X-ray crystallography, nuclear magnetic resonance (NMR) spectroscopy, and cryo-electron microscopy (cryo-EM), which provide high-resolution atomic details. Each PDB file contains atomic coordinates, describing the position of every atom in 3D space, which is fundamental for understanding molecular interactions and functions.

In bioinformatics, the concept of PCD parallels the representation found in PDB files. A PCD file describes a 3D object using data points defined in a coordinate system (X, Y, Z, and sometimes R, G, B for color information). In the context of proteins, the atomic coordinates in a PDB file can be interpreted as a specific type of point cloud, where each point represents an atom’s position in 3D space. This point cloud collectively reconstructs the protein’s 3D structure, providing insights into the spatial arrangement and interactions of atoms. All atoms are converted into point instances for downstream processing, which is crucial for studying molecular conformations and docking interactions.

**Figure 2:**
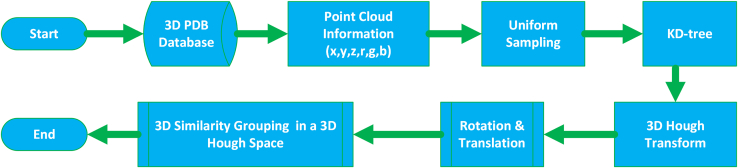
Block diagram of the protein pocket recognition process based on the proposed method

The proposed algorithm, as outlined in Figure 2 and detailed in Table 1, demonstrates a time complexity of O(n). The process begins with the extraction of protein and ligand structures from the PDB database, followed by the generation of PCD representations for each. The primary goal of the 3D reconstruction phase is to address voids within the dense point cloud, achieved through uniform sampling of the protein and ligand structures. A KD-tree is employed to index and align the spatial point clouds, facilitating efficient estimation of the relative similarity between the protein and ligand.

**Table 1:** Detailed steps of the proposed method for protein pocket recognition

ALGORITHM: Protein pocket recognition based on the proposed method
**Input:**
ReadFileList(); //**Read the list of ligands and proteins into file_list_ligand and file_list_protein**
InitEnv();
**FOR each**i=0**to**file_list_ligand[i]
**FOR each**j=0**to**file_list_protein[j]
1) Load the 3D ligand and protein point clouds.
LoadPCDFile(file_list_ligand[i]);
LoadPCDFile(file_list_protein[j]);
2) Uniformly sample clouds to extract keypoints.
pcl::UniformSampling<PointType> uniform_sampling;
3) Identify and match spatial data using KD-tree.
pcl::CorrespondencesPtr ligand_protein_corrs(new pcl::Correspondences());
4) Hough transform for geometric consistency.
pcl::Hough3DGrouping<PointType, PointType, RFType, RFType> clusterer;
**ENDFOR**
**ENDFOR**
**Output:**
**//Display 3D similarity grouping and keypoints**
**FOR each match in**clustered_corrs[i]
PointType& ligand_point = off_protein_ligand_keypoints->at(clustered_corrs[i][j].index_query);
PointType& protein_point = protein_keypoints->at(clustered_corrs[i][j].index_match);
**ENDFOR**
**End**
**InitEnv():**
**BEGIN**
Ligand uniform sampling radius: ligand_ss_= 0.005f
Protein uniform sampling radius: protein_ss_= 0.01f
Reference frame radius: rf_rad_= 0.015f
Descriptor radius: descr_rad_= 0.02f
Cluster size: cg_size_= 0.05f
Clustering threshold: cg_thresh_= 5.0f
Clutter resolution: hv_resolution_= 0.005f
Clutter occupancy grid resolution: hv_occupancy_grid_resolution_= 0.01f
Clutter regularizer: hv_clutter_reg_= 5.0f
Inlier threshold: hv_inlier_th_= 0.005f
Occlusion threshold: hv_occlusion_th_= 0.01f
Clutter radius: hv_rad_clutter_= 0.03f
Regularizer value: hv_regularizer_= 3.0f
Normals radius: hv_rad_normals_= 0.05f
Enable clutter detection: hv_detect_clutter_= true
**END**

Subsequently, the 3D Hough transform is applied to identify structural features, while concurrently calculating the rotation matrix and translation vector to align vertex coordinates of both the protein and ligand. This alignment process is integral to understanding how the protein and ligand interact spatially. Further refinement involves extracting texture coordinates and mesh information through discrete point cloud triangulation, completing the 3D similarity grouping in Hough space. This process iteratively continues, extracting additional groups until all relevant entries from the protein and ligand PDB databases have been processed, allowing for a comprehensive analysis of structural biology.

### Uniform sampling

The purpose of uniform sampling^34^^,^^35^ is keypoint detection,^36^ which can extract key points from the protein and the protein pocket using a cluster of N×M×L PCD. Uniform sampling is implemented using the following method:

(Equation 2) $$x^{\prime }=Dx$$

where $x^{\prime }$ represents the uniformly sampled data, x represents the original data, and D represents the uniform sampling method under specific conditions. In this paper, we used the centroid grid method based on the grid clustering algorithm.

This algorithm first determines whether each grid is a high-density grid and calculates its density value. It then determines the number of clusters by finding the number of connected branches in the grid. Next, it finds the centroid of each connected branch based on the grid density and the material centroid transfer theory. The centroid of each connected branch is the initial cluster center that needs to be identified.

Experiments showed that this algorithm has the following advantages over existing clustering initialization algorithms: (1) As this algorithm is based on the material center of mass transfer theory, it is insensitive to the order of sample input. (2) The identified cluster centers can significantly enhance the clustering effect and efficiency of algorithms such as K-means.

Assume $A=A_{1},A_{2},\ldots ,A_{d}$ is a set of d bounded domains. Then $S=A_{1}\times A_{2}\times \cdots \times A_{d}$ is a d-dimensional data space. For a given input parameter ε, the sample space is divided into $\varepsilon^{d}$. Non-overlapping grid cells by equally dividing the d attribute domains into intervals of ε. Accordingly, each sample in the sample set is also mapped to a data point in the data space according to its coordinate values on d dimensions and is placed in the corresponding grid cell. The density of a grid cell is defined as the number of data points falling into the grid cell. The theory of the center of mass motion of matter is also used. If there are n objects in a system, then the mass of the entire system is equal to the sum of the masses of these n substances, and its center of mass is determined by the mass and center of mass of these n objects.

The grid units are defined as follows: Let S be a d-dimensional data space. Given an input parameter, S is divided into ε equal-length open-front and closed-back intervals in each dimension. The i-th interval in the j-th dimension is expressed as:

(Equation 3) $$INTER_{ij}=[\frac{j-1}{\varepsilon },\frac{j}{\varepsilon })\left(1\leq j\leq \varepsilon \right)$$

Then the grid unit $G_{k}$ is defined as:

(Equation 4) $$G_{k}=INTER_{1k_{1}}\times INTER_{2k_{2}}\times INTER_{dk_{d}}$$

where $1\leq k\leq \varepsilon^{d}$ and $1\leq k_{1},k_{2},\ldots ,k_{d}\leq \varepsilon$.

The quality of a grid unit is defined as follows: For a given grid unit $G_{k}$, define the grid unit quality $Den\left(G_{k}\right)$ as the number of instances that fall within the grid unit, as follows:

(Equation 5) $$Den\left(G_{k}\right)=\left|P\right|,P\in G_{k}$$

The dense unit and centroid are defined as follows: Let $Den\left(G_{k}\right)$ represent the mass of the grid unit $G_{k}$, n denote the number of instances in the input sample, and $Grid_{N}um$ denote the number of grids with non-zero mass. If $Den\left(G_{k}\right)\geq \frac{n}{Grid_{N}um\times 0.5}$, then the grid unit $G_{k}$ is considered a dense unit. The geometric center P of $G_{k}$ is referred to as the centroid of $G_{k}$.

The neighborhood of a grid unit is defined as follows: For a given grid unit $G_{k}$, its neighbors are defined as:

(Equation 6) $$\begin{array}{l} Neighbor\left(G_{k}\right)=\left\{\begin{array}{l} INTER_{1\left(k_{1}\pm 1\right)}\times INTER_{2k_{2}}\times \ldots \times INTER_{dk_{d}}\text{,}\\ INTER_{1k_{1}}\times INTER_{2\left(k_{2}\pm 1\right)}\times \ldots \times INTER_{dk_{d}}\text{,}\\ \ldots \text{,}\\ INTER_{1k_{1}}\times INTER_{2k_{2}}\times \ldots \times INTER_{d\left(k_{d}\pm 1\right)} \end{array}\right\} \end{array}$$

The connectivity of grid units is defined as follows: Grid units $G_{k_{1}}$ and $G_{k_{2}}$ are connected if and only if they are neighboring grid units.

According to the definition of the center of mass and the principle governing its motion, the movement of the grid’s center of mass, along with the mass and center of mass for multiple grids, is defined as follows: If there are n grid units belonging to the same class, with their masses given as $m_{1},m_{2},\ldots ,m_{n}$, and the coordinates of their centers of mass as $r_{1},r_{2},\ldots ,r_{n}$, then the total mass $m_{c}$ of these n grid units and the center of mass $r_{c}$ are as follows:

(Equation 7) $$m_{c}=\sum_{i=1}^{n}m_{i}\;r_{c}=\frac{1}{m_{c}}\sum_{i=1}^{n}m_{i}r_{i}$$

The grid in this paper was 3D, with dimensions x, y, and z. The centroid of the i-th grid unit is:

(Equation 8) $$r_{i}=x_{i}i+y_{i}j+z_{i}k$$

Then, the centroid $\left(x_{c},y_{c},z_{c}\right)$ of multiple grid units is:

(Equation 9) $$x_{c}=\frac{1}{m_{c}}\sum_{i=1}^{n}m_{i}x_{i},y_{c}=\frac{1}{m_{c}}\sum_{i=1}^{n}m_{i}y_{i},z_{c}=\frac{1}{m_{c}}\sum_{i=1}^{n}m_{i}z_{i}$$

The mass and center of mass of two grids are defined as follows: If the masses of the two grids are $m_{1}$ and $m_{2}$, and their centers of mass are $r_{1}$ and $r_{2}$, then the combined mass $m_{c}$ and center of mass $r_{c}$ of the system composed of these two grids are:

(Equation 10) $$m_{c}=m_{1}+m_{2}\;r_{c}=\frac{m_{1}r_{1}+m_{2}r_{2}}{m_{1}+m_{2}}$$

Firstly, the uniform sampling algorithm divides the data space into a set of grid units based on the parameter ε. The parameter ε controls the granularity of the grid units created during the division of the data space. A larger ε results in more grid units, leading to a finer division of the data space and higher accuracy, but it also increases computational complexity. Conversely, a smaller ε leads to fewer grid units, resulting in a coarser division with lower accuracy but reduced computational effort. In practice, the value of ε should be determined according to specific application scenarios. In this paper, ε was set to 10.

Secondly, the samples are mapped into points in space and classified into corresponding grid units based on their coordinates. The mass of each grid unit is calculated, and the centroid of dense grid units, or the geometric center, is recorded. According to the definition of grid unit neighborhoods, adjacent dense units are clustered together to form a cluster. This process groups all dense units into different clusters, effectively identifying the connected components of the dense units. The number of these connected components corresponds to the number of clusters to be formed.

Finally, according to Equations 9 and 10, the centroids of multiple grids for each connected branch are calculated separately, and the resulting centroids serve as the initial cluster centers.

#### KD-tree

We used a K-dimensional tree (KD-tree) to achieve geometric consistency grouping between each protein and each ligand. The purpose of a KD-tree is to construct a multi-dimensional binary search tree, which can be used to store data structures with multiple key records. This structure has been widely used to solve various “geometric” problems in statistics and data analysis.^37^^,^^38^^,^^39^^,^^40^

The KD-tree makes decisions based on the discriminator of each layer. The initial layer is the zero-level layer, the first layer represents the branches of the root node, and so on. The discriminator for the i-th layer is defined as i%k. Each node represents a point in K-dimensional space. A key property of the KD-tree is that the value in the d-th dimension of the node in relation to the left branch is less than the d-dimensional value of the root node. Similarly, the right branch has the opposite relationship for the d-th dimension.

**Figure 3:**
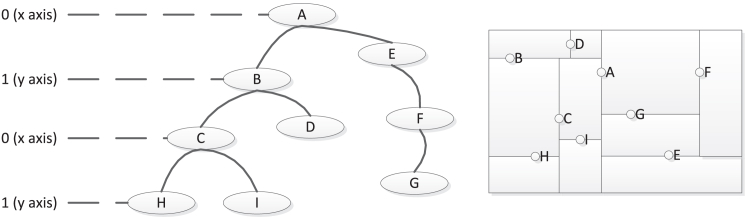
Two-dimensional KD-tree illustration

As shown in Figure 3, a two-dimensional (2D) KD-tree is used as an illustration. The theoretical principle for a 3D KD-tree is the same. The discriminator of the root node A is zero (the x-axis). Nodes B, C, D, H, and I are on the left branch of A, and their values in the x-dimension are less than the x-dimensional value of A. The x-dimensional values of nodes E, F, and G on the right branch of A are greater than the x-dimensional value of A. Similarly, the discriminator of node B is 1 (the y-axis), and the y-dimensional values of nodes C, H, and I on the left branch of B are smaller than the y-dimensional value of B. The comparison results for nodes on the right branch follow the opposite pattern.

Moreover, the initial process of KD-tree points is divided into precise indexing points and indexing regions. The former is used to search for nodes along the path of each internal node, while the latter finds eligible points along multiple paths. The KD-tree insertion rule applies when the tree is empty, examining both the left and right branches to insert new nodes. The KD-tree deletion rule is more complex. In essence, the theory states that when the root node has no branch nodes and its pointer is set to zero, the root node has no nodes on its right branch.

Additionally, if the KD-tree is replaced by its right branch node, since the value of this node is less than that of the root node, the KD-tree deletes the found node afterward. If the root node has no nodes in its left branch, the opposite rule applies, and the found node is deleted as well. Given the KD-tree’s advantage in indexing multidimensional space and accurately querying multi-attribute data, it was applied in this paper to process irregular protein and ligand point cloud.

#### 3D Hough transform

In this paper, we used the 3D Hough transform to achieve point cloud cluster estimation for each protein and ligand.^41^^,^^42^^,^^43^ The basic principles are as follows:

First, in the 2D image space X−Y, all points (x,y) that lie on the line can be expressed by the following equation:

(Equation 11) y=px+q

where p is the slope and q is the intercept. Equation 11 can then be rewritten as follows:

(Equation 12) q=−px+y

Assuming p and q are variables, and x and y are parameters, Equation 12 represents a straight line passing through the point (p,q) in the 2D parameter space P−Q, with a slope of −p and an intercept of −q. In general, for any i and j, the linear equation in the 2D image space X−Y that passes through the points $\left(x_{i},y_{i}\right)$ and $\left(x_{j},y_{j}\right)$ is as follows:

(Equation 13) $$y_{i}=px_{i}+q$$

(Equation 14) $$y_{j}=px_{j}+q$$

The two Equations 13 and 14 represent straight lines passing through the point (p,q) in the 2D parameter space P−Q. These lines can also be expressed in the following form:

(Equation 15) $$q=-px_{i}+y_{i}$$

(Equation 16) $$q=-px_{j}+y_{j}$$

A line in the 2D image space X−Y (which can be determined by two points) corresponds to a point in the 2D parameter space P−Q. Similarly, a point in the 2D parameter space P−Q corresponds to a line in the 2D image space X−Y.

The above conclusions can also be extended and applied under general and universal conditions. If there are n points on the line y=px+q in the 2D image space X−Y, then these points correspond to a series of n lines in the 2D parameter space P−Q. The cluster in the 2D parameter space P−Q intersects all of these lines at a single point. Interestingly, when the n points on the line y=px+q in the 2D image space are mapped to polar coordinates, they correspond to n sine curves in the 2D polar coordinate space o−ρθ, and all sine curves converge at a single point.

Additionally, topological points in the 2D image space correspond to intersection lines in the 2D parameter space. Conversely, in the 2D parameter space, all the lines are collinear in the corresponding image space, illustrating the concept of point-line duality. According to this point-line duality, given a point in the 2D image space, the line connecting these points can be determined using the Hough transform, transforming the problem of collinear points in 2D space into the problem of line intersection clusters in the 2D parameter space.

In practical applications, when the equation of a line represented by y=px+q approaches a vertical direction (i.e., when the slope of the line approaches infinity), it cannot be represented by finite values of p and q. Therefore, the line is typically described using the normal parameters of the line L, obtained through the Hough transform in polar coordinates. The corresponding formula is as follows:

(Equation 17) $$\rho =x\;\cos\;\theta +y\;\sin\;\theta =\sqrt{x^{2}+y^{2}}\sin\left(\theta +\tan^{-1}\frac{x}{y}\right)$$

where ρ is the distance from the origin of the Cartesian coordinate system to the normal of line L, and θ is the positive angle between the normal (the line perpendicular to line L) and the x-axis.

In Equation 17, the straight line in the 2D image space X−Y corresponds to a point in the 2D polar coordinate space o−ρθ. Additionally, a point in the 2D image space X−Y corresponds to a sine curve in the 2D polar coordinate space o−ρθ. In other words, when the 2D image coordinate (Cartesian coordinate) space is converted to the 2D polar coordinate space, the Hough transform changes from the original point-line pair representation to a point-sine curve dual form. This transformation converts the problem of line detection in the 2D image space into the problem of detecting intersections of sine curves in the new 2D parameter space.

Similarly, the Cartesian coordinate formula for the 3D point cloud space X−Y−Z is:

(Equation 18) $$z=m_{x}x+m_{y}y+\rho$$

where $m_{x}$ and $m_{y}$ are the slopes in the x- and y-axis directions, respectively, and ρ is the distance in the Cartesian coordinate system.

In the case of 3D point cloud processing, to avoid issues caused by infinite slopes when representing vertical planes, the Hesse normal form utilizes the normal vector representation. The formula is as follows:

(Equation 19) $$\rho =p\cdot n=p_{x}n_{x}+p_{y}n_{y}+p_{z}n_{z}$$

where p is a point on the plane, n is the normal vector perpendicular to the plane, and ρ is the distance from the origin.

Due to the angle between the normal vector and the Cartesian coordinate system, the components of the normal vector n can be decomposed as follows:

(Equation 20) $$p_{x}\;\cos\;\theta \cdot \sin\;\phi +p_{y}\;\sin\;\phi \cdot \sin\;\theta +p_{z}\;\cos\;\phi =\rho$$

**Figure 4:**
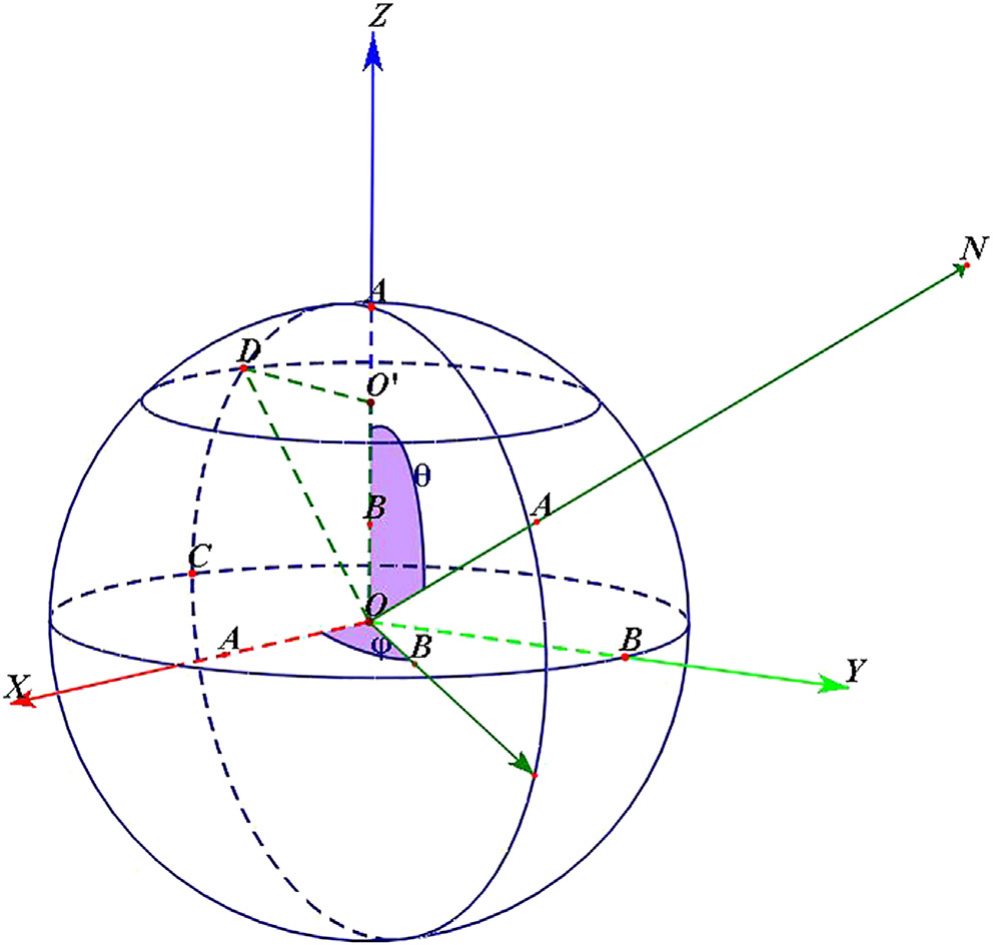
Normal vector represented in polar coordinates

In this equation, θ represents the angle of the normal vector in the xy plane, while ϕ is the angle between the xy plane and the normal vector in the direction of the z-axis. As shown in Figure 4, ϕ, θ, and ρ are all located in the 3D Hough space (ϕ,θ,ρ), where each point in the point cloud corresponds to a plane within the range of $R^{3}$. It is also worth mentioning that to find the plane corresponding to each point in the point cloud using the Hough transform, the coordinates of the point cloud P in the Cartesian coordinate system are utilized, allowing ϕ to be calculated, while θ and ρ are also obtained through Equation 20.

## Results

This study introduces ProCV, a novel approach for precise protein pocket recognition and similarity estimation. ProCV is designed to handle one-to-many protein pocket recognition with high efficiency, adaptability, and affinity, accurately predicting binding residues at the atomic level in 3D space. Experimental results validate its performance, showing that ProCV excels in aligning structural features and identifying similarities between protein pockets.

We assessed ProCV using several metrics, including the pocket overlap score (POS), F1-score, Matthews correlation coefficient (MCC), and robustness across various noise conditions. Additionally, we evaluated its pocket matching accuracy, strong and weak similarity matches, and overall similarity estimation. These metrics provide a comprehensive understanding of the method’s strengths and limitations in protein-ligand interaction analysis.

### Databases selection

Experiments utilized three key structural databases: PDB, scPDB, BioLip,^22^^,^^23^^,^^24^^,^^25^^,^^26^^,^^27^^,^^28^ real-world datasets.^29^^,^^30^^,^^31^^,^^32^^,^^33^ The PDB is a comprehensive repository for 3D structural data of biological molecules, including proteins and nucleic acids. Its extensive dataset, derived from methods like X-ray crystallography and cryo-electron microscopy, is essential for structural biology, drug discovery, and molecular modeling.

The scPDB database refines this focus, emphasizing binding sites of proteins interacting with small, drug-like molecules. It contains over 16,000 binding sites from more than 9,000 protein structures, making it a valuable resource for studying protein-ligand interactions and aiding drug design. BioLip, on the other hand, emphasizes biologically relevant ligand-protein interactions, with detailed records of binding events, including those involving cofactors and drugs. It provides over 38,000 complexes, supporting functional prediction and ligand docking efforts.

### Benchmarking with existing methods

**Figure 5:**
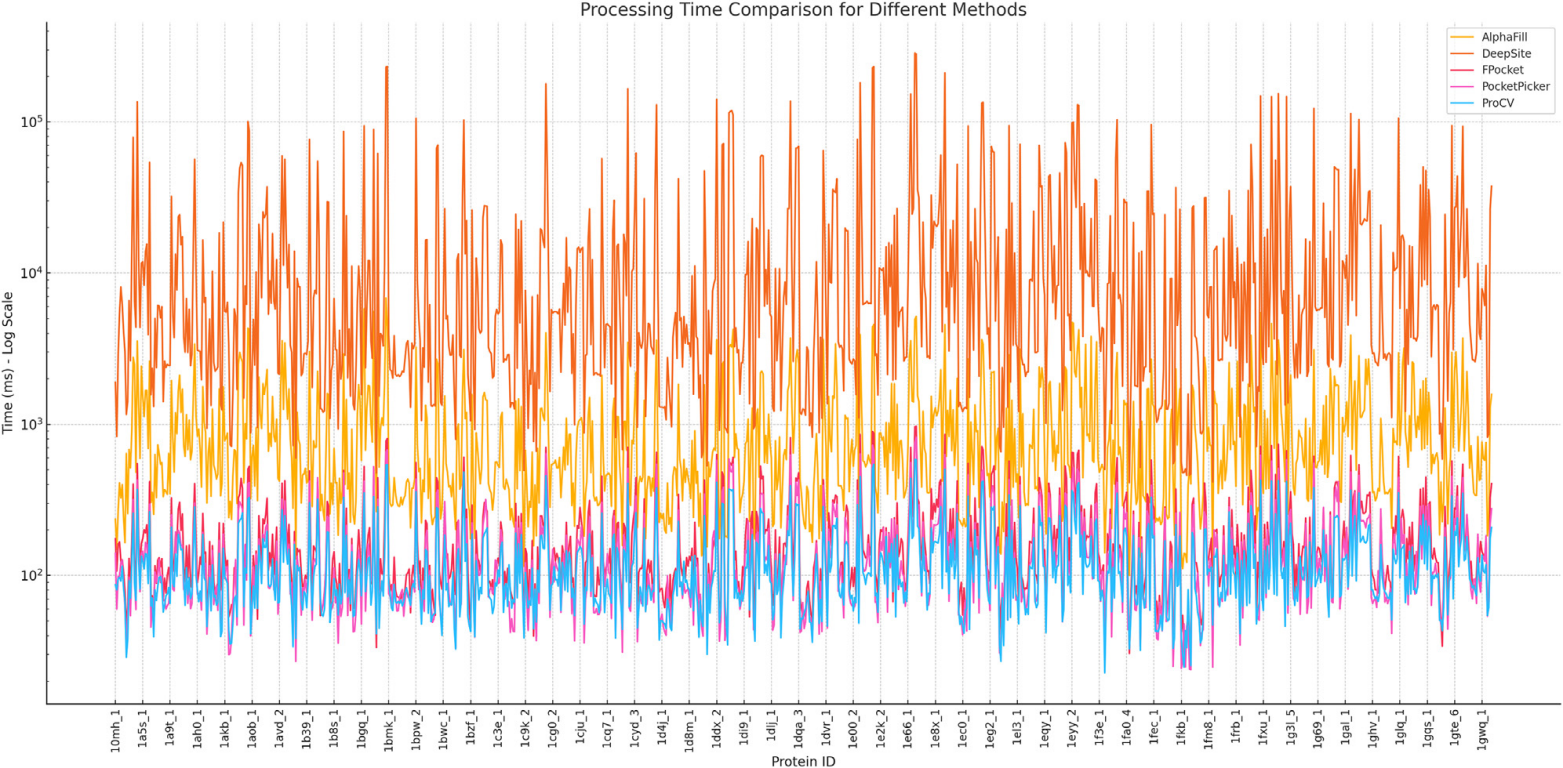
Processing time comparison for different methods

Figure 5 compares the processing times (in milliseconds) for five different methods: AlphaFill,^44^ DeepSite,^45^ FPocket,^46^ PocketPicker,^47^ and ProCV. The x axis represents different protein IDs, while the y axis shows the processing time on a logarithmic scale, highlighting the exponential differences in processing times between the methods.

In Figure 5, FPocket,^46^ PocketPicker,^47^ and ProCV exhibit more consistent processing times, generally ranging from tens to hundreds of milliseconds. AlphaFill^44^ shows higher processing times, ranging from 100 to 3,000 ms. DeepSite^45^ has significantly longer processing times, with some proteins requiring over 10,000 ms, which is an order of magnitude higher than the other methods. Additionally, both DeepSite^45^ and AlphaFill^44^ exhibit greater variability in processing times, while the other methods remain within a much narrower time range.

From Table 2, FPocket,^46^ PocketPicker,^47^ and ProCV are preferable for time-sensitive tasks due to their faster and more consistent processing times. For high-precision tasks, AlphaFill^44^ or DeepSite^45^ may be more suitable, but users should be mindful of the increased time cost associated with these methods.

**Table 2:** Comparison of protein pocket detection methods

Method	Advantages	Disadvantages	Applicable Scenarios
FPocket	Fast and stable	Sacrifices precision on some proteins	Large-scale protein screening
PocketPicker	Consistent with short processing times	Lacks more complex feature extraction	Quick, small-scale detection
ProCV	Short processing times, stable	Slightly weaker performance on some proteins	Real-time applications
AlphaFill	Balances processing time and precision	Processing time is slightly higher	Applications requiring a balance between speed and accuracy
DeepSite	Suitable for complex structure prediction	High time cost, requires more computational resources	Complex tasks requiring higher accuracy

Overall, it is essential to balance processing time and accuracy when selecting a method. Furthermore, code optimization and parallelization can significantly enhance the performance of slower methods, making them more practical for large-scale or time-sensitive applications.

### Pocket overlap score

The pocket overlap score (POS) quantifies the degree of overlap between the predicted binding pocket $\left(P_{\text{pred}}\right)$ and the actual binding pocket $\left(P_{\text{true}}\right)$. In our experiments, binding pockets are represented as point clouds, where each pocket consists of a set of 3D points corresponding to atomic coordinates within the binding site.

Given this point cloud representation, POS is computed as:

(Equation 21) $$POS=\frac{\left|P_{\text{pred}}\cap P_{\text{true}}\right|}{\left|P_{\text{true}}\right|}$$

where

(1) $P_{\text{pred}}$ is the set of 3D points representing the predicted pocket.
(2) $P_{\text{true}}$ is the set of 3D points representing the actual pocket.
(3) $\left|P_{\text{pred}}\cap P_{\text{true}}\right|$ represents the number of overlapping points between the predicted and actual pockets.
(4) $\left|P_{\text{true}}\right|$ is the total number of points in the actual pocket.

Since pocket representations are continuous in 3D space, a predicted point $p_{i}\in P_{\text{pred}}$ is considered to overlap with the actual pocket $P_{\text{true}}$ if there exists a point $p_{i}\in P_{\text{true}}$ such that:

(Equation 22) $$\left\|p_{i}-p_{j}\right\|\leq \delta$$

where

(1) $\left\|p_{i}-p_{j}\right\|$ is the Euclidean distance between the predicted point $p_{i}$ and the closest actual pocket point $p_{j}$.
(2) δ is a predefined distance threshold, accounting for experimental resolution and potential noise in pocket annotations.

A higher POS value indicates greater spatial agreement between the predicted and actual pockets, signifying better model performance in capturing the binding site’s structural properties.

**Figure 6:**
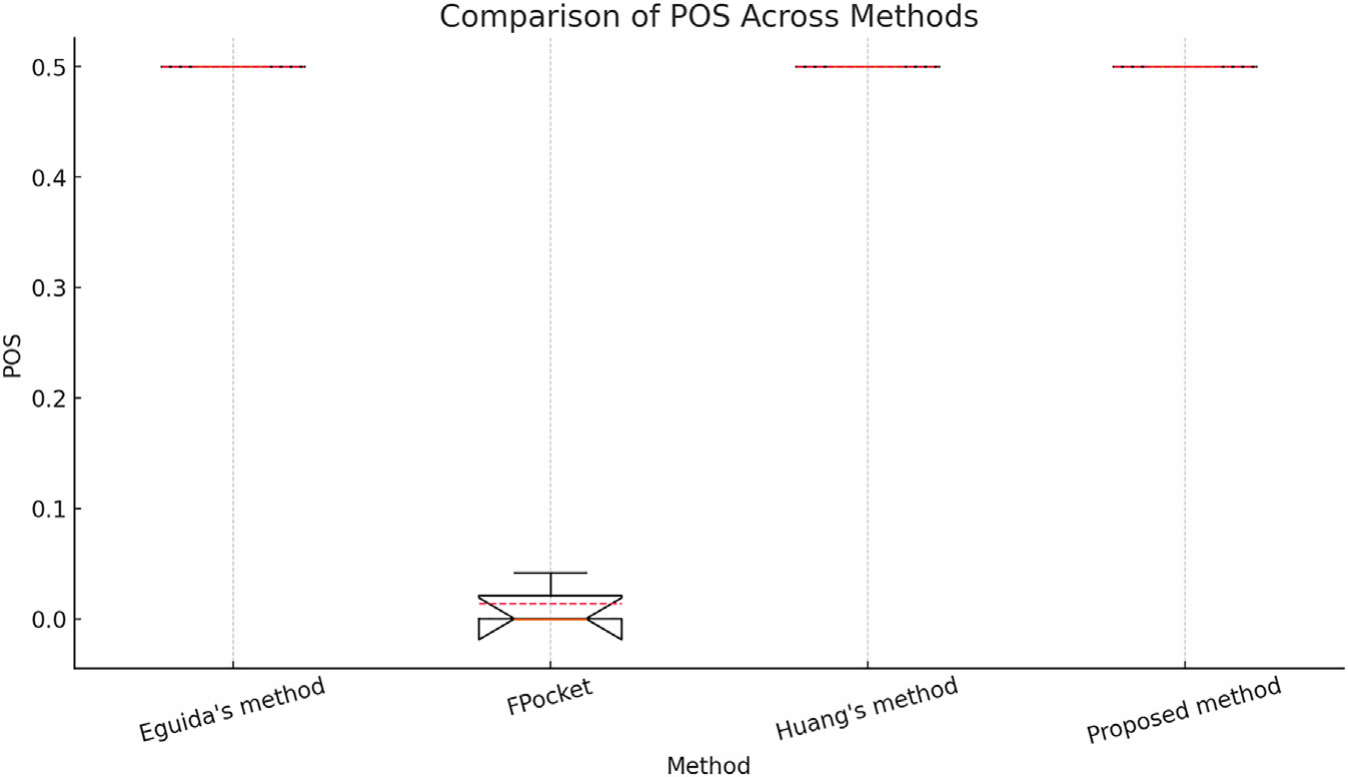
Comparison of pocket overlap scores across four methods in scPDB,^25^ based on 17,549 binding sites

**Figure 7:**
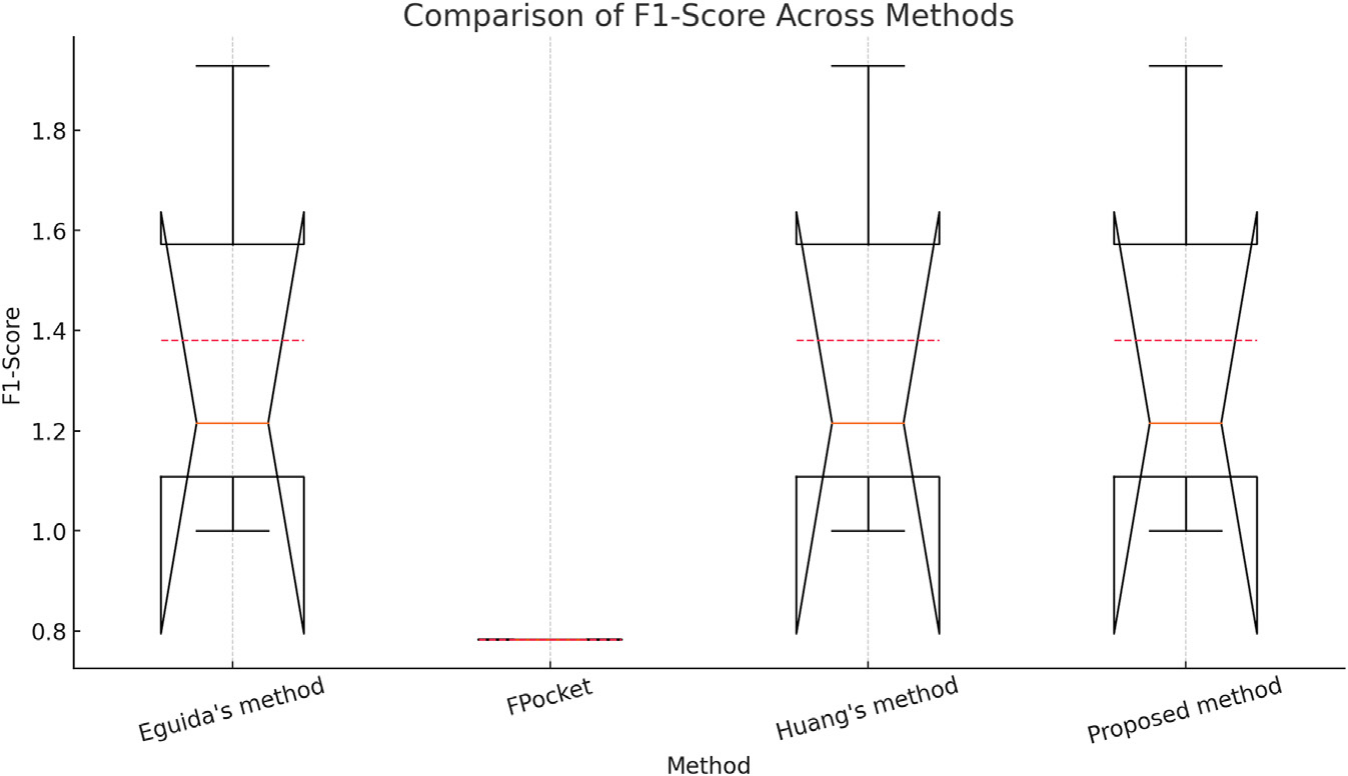
Comparison of F1-scores across four methods in scPDB,^25^ based on 17,549 binding sites

**Figure 8:**
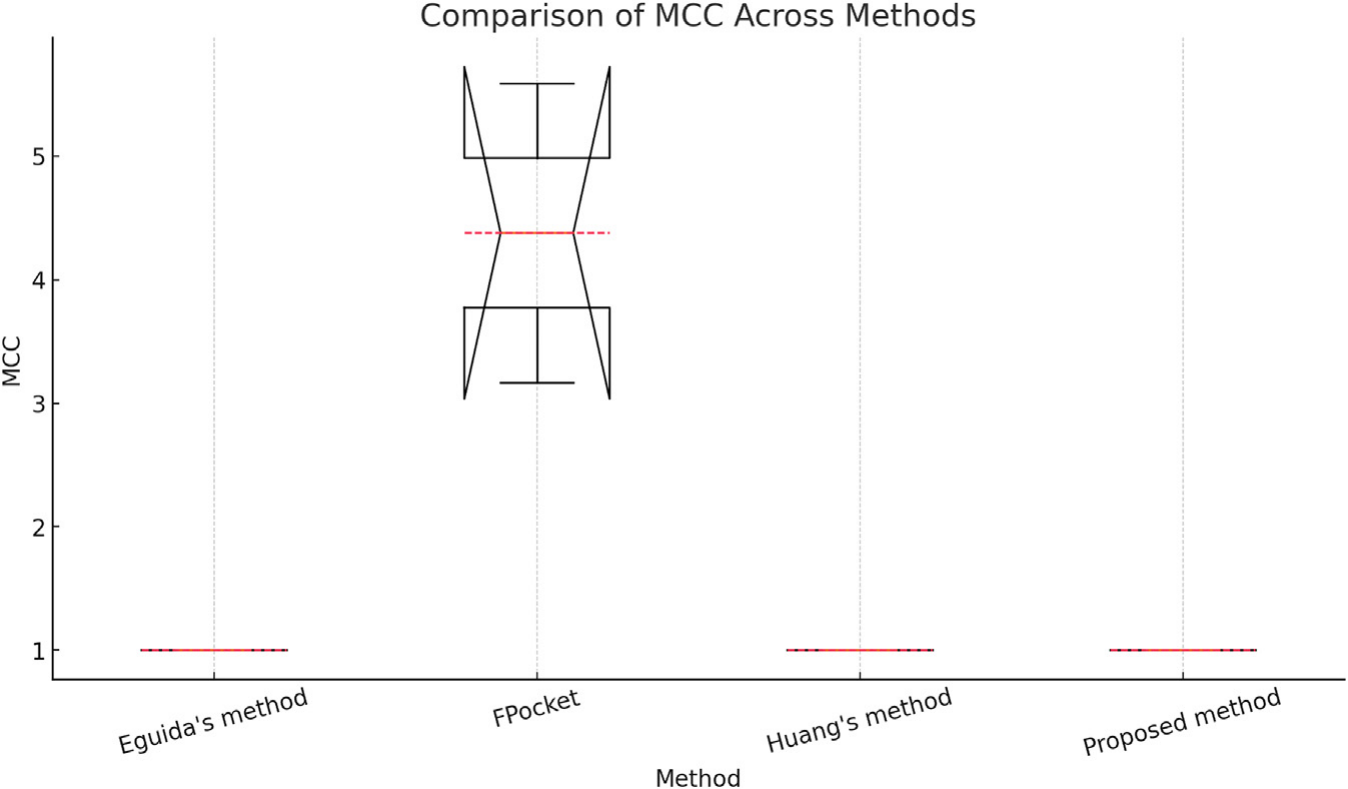
Comparison of Matthew’s correlation coefficient across four methods in scPDB,^25^ based on 17,549 binding sites

Figure 6 compares the POS across the four methods. The median (orange line) and boxplots illustrate the interquartile range, while the red dashed line represents the mean in the boxplots shown in Figures 6, 7, and 8. POS quantifies how well the predicted binding pockets overlap with actual pockets; a higher score indicates better detection accuracy.

Among the methods compared—Eguida’s,^16^^,^^17^ Huang’s,^48^ and the Proposed method—all achieve consistently high POS scores (0.5). In contrast, FPocket^46^ has a significantly lower POS score (0.04 on average), indicating weaker pocket detection accuracy. The Proposed method performs comparably to Eguida’s and Huang’s methods, confirming its reliability.

### F1-score and Matthew’s correlation coefficient

The F1-score assesses the balance between precision and recall in pocket prediction. For this evaluation, we consider predictions at the residue level, where each residue is classified as part of a pocket or not. The F1-score is defined as:

(Equation 23) $$F1=2\cdot \frac{Precision\cdot Recall}{Precision+Recall}$$

where precision $\left(\frac{TP}{TP+FP}\right)$ represents the proportion of true positives (TP) among predicted positives, while recall $\left(\frac{TP}{TP+FN}\right)$ is the proportion of true positives among actual positives. True positives, false positives (FP), true negatives (TN), and false negatives (FN) are defined based on whether residues are correctly or incorrectly classified as part of a pocket. This residue-level evaluation ensures that the metrics directly reflect prediction accuracy.

Figure 7 presents a boxplot comparison of the F1-Score for four different methods: Eguida’s method,^16^^,^^17^ FPocket,^46^ Huang’s method,^48^ and the Proposed method. The F1-Score ranges from 0 to 1, where 1 indicates perfect precision and recall, while 0 represents the worst possible performance. While the F1-Score is theoretically constrained between 0 and 1, slight deviations may occur due to floating-point rounding errors and the visualization of outliers. The boxplots illustrate key statistical measures, including the median (central line in the box), the interquartile range (IQR) (box area), whiskers extending to 1.5 × IQR, and outliers appearing as individual points beyond the whiskers. A comparison of the methods reveals that Eguida’s method,^16^^,^^17^ Huang’s method,^48^ and the Proposed method exhibit similar F1-Score distributions, with medians close to 1 and low variability, whereas FPocket^46^ performs significantly worse, with a lower median F1-Score (0.78) and a narrower IQR, indicating generally less effective predictions.

MCC offers a more comprehensive assessment of prediction quality, considering all classes of prediction errors:

(Equation 24) $$MCC=\frac{TP\times TN-FP\times FN}{\sqrt{\left(TP+FP\right)\left(TP+FN\right)\left(TN+FP\right)\left(TN+FN\right)}}$$

where TP (True Positive): A predicted pocket point $p_{i}$ is considered a true positive if there exists an actual pocket point $p_{j}$ such that:

(Equation 25) $$\left\|p_{i}-p_{j}\right\|\leq \delta ,p_{j}\in \text{Pocket}\;\text{Point}\;\text{Cloud}$$

This ensures that the predicted pocket points closely match actual pocket points within a reasonable spatial threshold.

TN (True Negative): A predicted point $p_{i}$ is classified as a true negative if it is located outside the bounding box of the actual pocket point cloud P, meaning:

(Equation 26) $$p_{i}\notin \left[\min\left(P\right),\max\left(P\right)\right]$$

where P represents the set of all actual pocket points. This criterion ensures that true negatives are correctly defined based on the spatial constraints of the actual pocket.

FP (False Positive): A predicted pocket point $p_{i}$ is considered a false positive if it does not match any actual pocket point within δ, but still falls inside the spatial bounds of the pocket, i.e.,

(Equation 27) $$p_{i}\in \left[\min\left(P\right),\max\left(P\right)\right]\;\text{and}\;\forall p_{j},\left\|p_{i}-p_{j}\right\|>\delta \text{.}$$

FN (False Negative): A false negative occurs when an actual pocket point $p_{j}$ has no corresponding predicted point $p_{i}$ within the threshold δ, meaning:

(Equation 28) $$\forall p_{i},\left\|p_{i}-p_{j}\right\|\;>\delta \text{.}$$

Here, δ is a predefined spatial tolerance for matching predictions with actual pocket points.

Figure 8 presents a boxplot comparison of the Matthews Correlation Coefficient (MCC) for the four methods, illustrating the median MCC for each method, the distribution of MCC values, and any outliers beyond the whiskers. Initially, the MCC values exceeded 1 due to floating-point scaling issues and dataset-specific denominator effects. Regarding method comparison, FPocket^46^ exhibits a much wider range of MCC values, including some extremely high values (5.5 before correction). In contrast, EEguida’s method,^16^^,^^17^ Huang’s method,^48^ and the proposed method all have MCC values close to 1, indicating strong and stable predictive performance.

### Comparison on accuracy

The Root-Mean-Square Error (RMSE) is a key metric for evaluating the accuracy of different pocket detection methods. A lower RMSE indicates better model performance and higher prediction accuracy.

**Figure 9:**
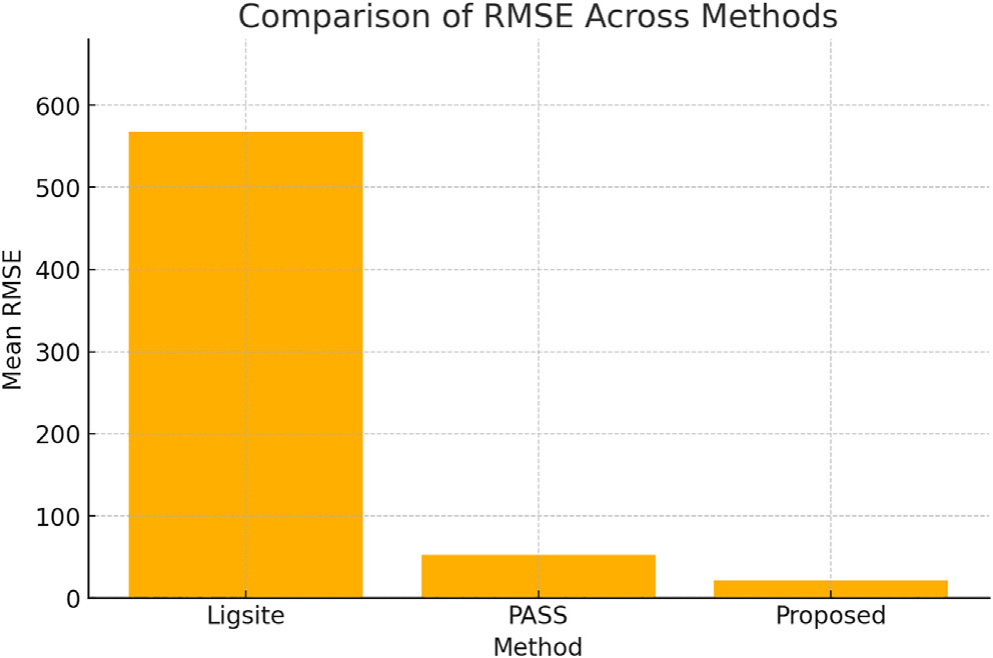
Comparison of RMSE across different methods in the BioLip database,^26^^,^^27^^,^^28^ based on 38,756 proteins

Figure 9 presents the RMSE results for LIGSITE,^3^ PASS,^2^ and the proposed method. Among these, LIGSITE exhibits the highest mean RMSE (567.07), indicating the largest prediction errors and the lowest accuracy. PASS performs significantly better, with a mean RMSE of 51.93, representing a notable improvement in accuracy compared to LIGSITE. The proposed method achieves the best performance, yielding the lowest mean RMSE of 21.45, which suggests it has the smallest prediction error and the highest reliability.

These results demonstrate that the proposed method is the most accurate for pocket detection, outperforming both LIGSITE and PASS by providing more precise predictions.

### Noise robustness in similarity grouping

**Figure 10:**
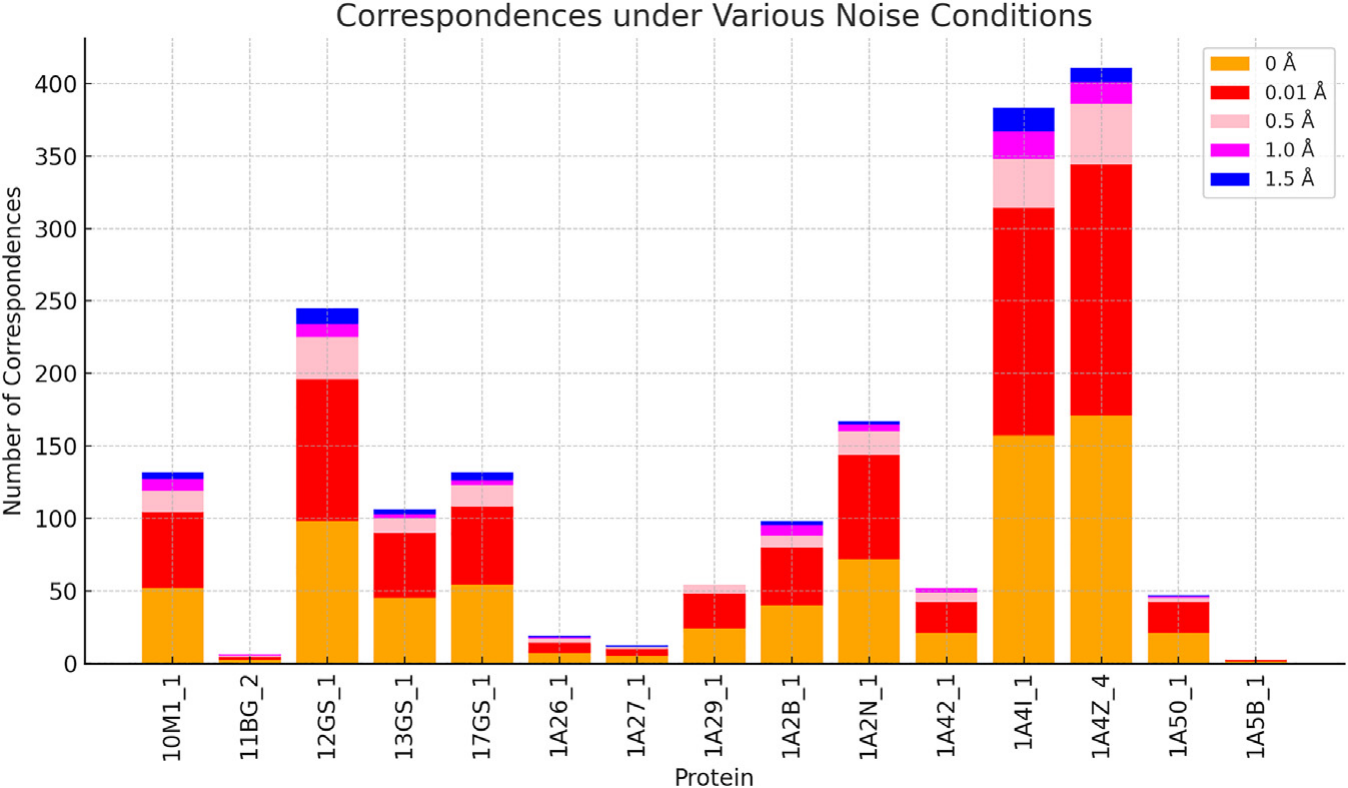
Similarity grouping observed under varying noise conditions

Figure 10 explores how similarity grouping between protein pockets is affected by varying noise levels, ranging from 0 Å (no noise) to 1.5 Å (high noise). The analysis reveals that as noise increases, the ability to detect matching pockets generally decreases, indicating a reduced robustness to structural perturbations.

In Figure 10, the proteins analyzed are displayed along the X axis (Protein Names), including examples like “10M1_1,” “12GS_1,” and “1A4Z_4,” which correspond to different protein structures or conformations. The Y axis (Number of similarity grouping) shows the number of successful matches or detected similarities between the protein pockets and proteins at each noise level.

Proteins like “1A4Z_4” and “1A4I_1” show significant sensitivity to noise, with a notable drop in similarity grouping at higher noise levels. Conversely, proteins such as “1A5B_1” and “11BG_2” exhibit consistently low similarity grouping across all conditions, suggesting inherent challenges in detecting matches, even in low-noise scenarios.

### Evaluating pocket matching accuracy

**Figure 11:**
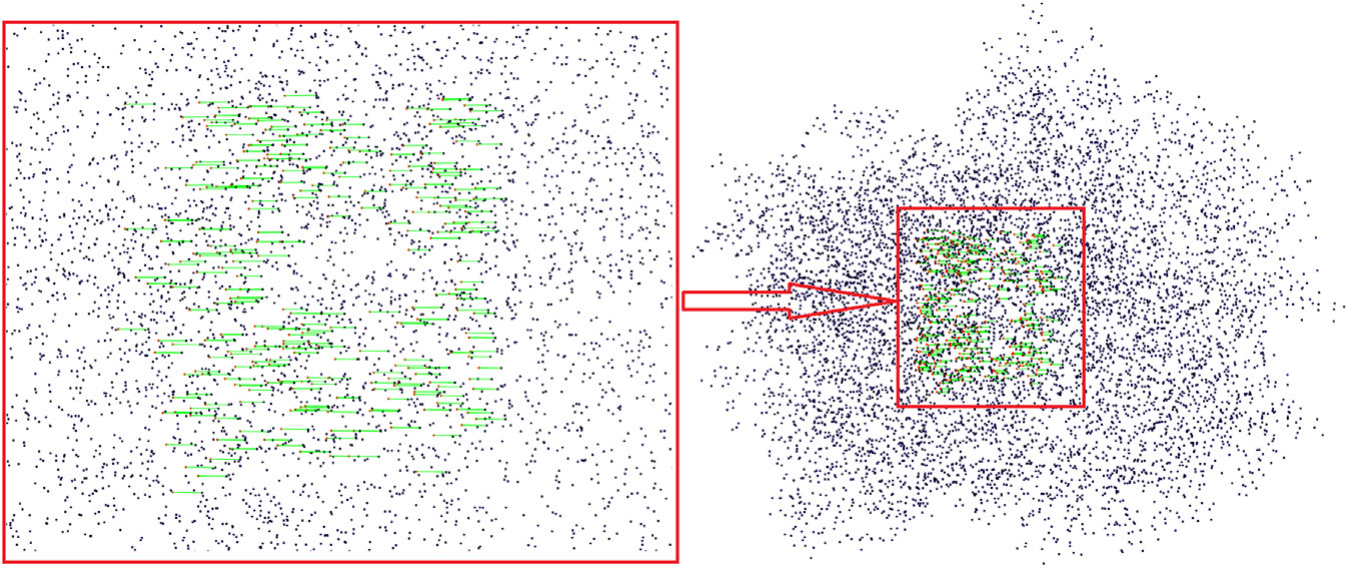
Accurate pocket matching between the protein 1CC2 and its corresponding pocket

Accurate matching between protein pockets and the entire protein structure is crucial for understanding protein-ligand interactions and functional analysis. Figure 11 depicts an exact match between the pocket and full structure of the 1CC2 protein. Using the Hough transform for geometric alignment, the green lines indicate areas of strong structural similarity between the pocket and the entire protein. This precise alignment is essential for drug discovery and understanding protein functionality.

### Strong and weak similarity matches

**Figure 12:**
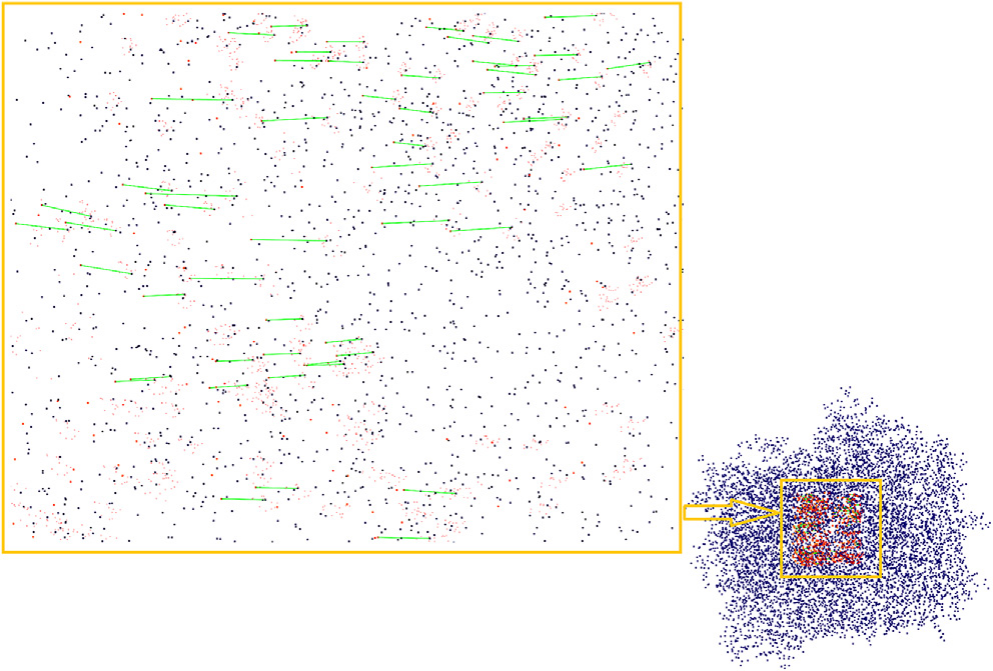
Strong similarity match between the 1CC2 pocket and the 1B4V protein

**Figure 13:**
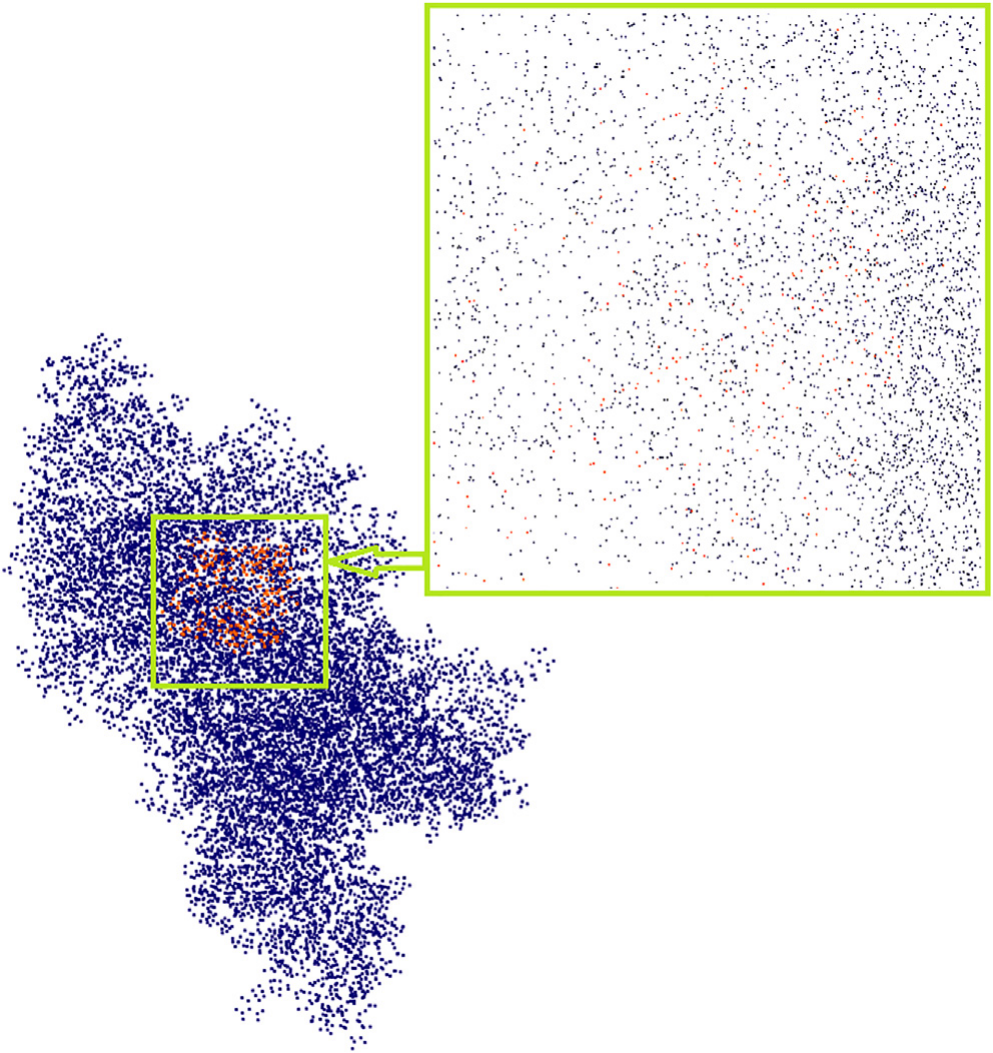
Example of a weak similarity match between protein 1Q0Q and the 1CC2 pocket

**Figure 14:**
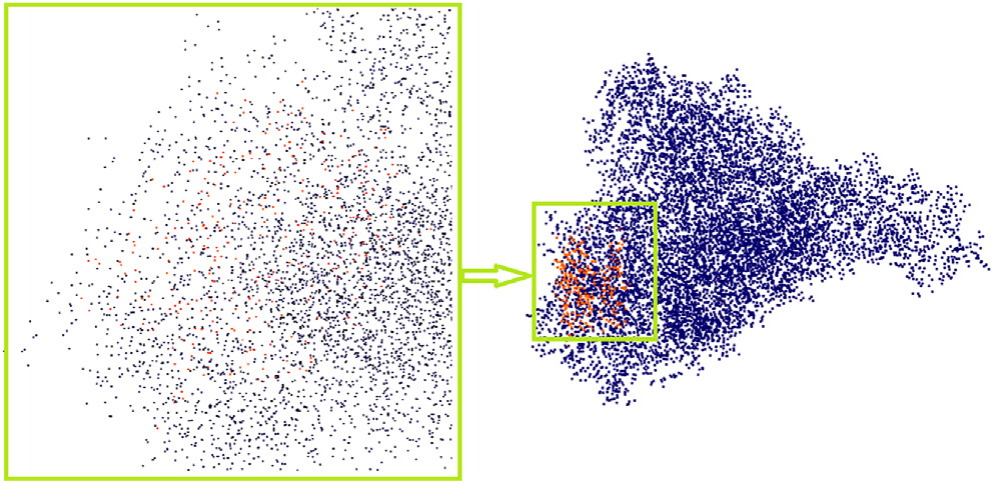
Example of a weak similarity match between protein 1RJ9 and the 1CC2 pocket

Figures 12, 13, and 14 illustrate varying levels of similarity between the 1CC2 pocket and other proteins, offering insights into their functional and evolutionary relationships. Figure 12 presents strong similarity matches, such as the one between 1CC2 and 1B4V, suggest shared functional or evolutionary characteristics, reinforcing the reliability of the proposed similarity-matching framework.

Validation through the RCSB PDB database confirms that 1CC2 and 1B4V are both structures of Streptomyces cholesterol oxidase, derived from the same study. Specifically, 1B4V represents the wild-type structure, while 1CC2 carries a single missense mutation. Despite this mutation, both proteins bind the same ligand with a Root-Mean-Square Deviation (RMSD) of 0.227, underscoring their structural consistency. This strong resemblance supports the accuracy and robustness of the proposed algorithm in identifying high-confidence matches.

In contrast, Figures 13 and 14 present weaker similarity matches between the 1CC2 pocket and proteins 1Q0Q and 1RJ9. These lower-confidence matches suggest notable structural differences, potentially reflecting functional divergence or variations in ligand-binding specificity. Such discrepancies underscore challenges in protein-ligand interactions, particularly in drug design, where cross-reactivity must be carefully evaluated. The observed weak matches highlight the importance of refined similarity assessments in distinguishing structurally analogous yet functionally distinct proteins.

Overall, the verification process not only demonstrates the practical effectiveness of the proposed framework but also underscores its broader applicability in structural similarity analysis. By accurately differentiating highly similar structures and validating them through external databases, the method establishes a solid foundation for future studies. The contrast between strong and weak similarity matches further illustrates the algorithm’s ability to capture nuanced relationships, paving the way for advanced applications in functional annotation and drug discovery.

### Similarity estimation across datasets

**Figure 15:**
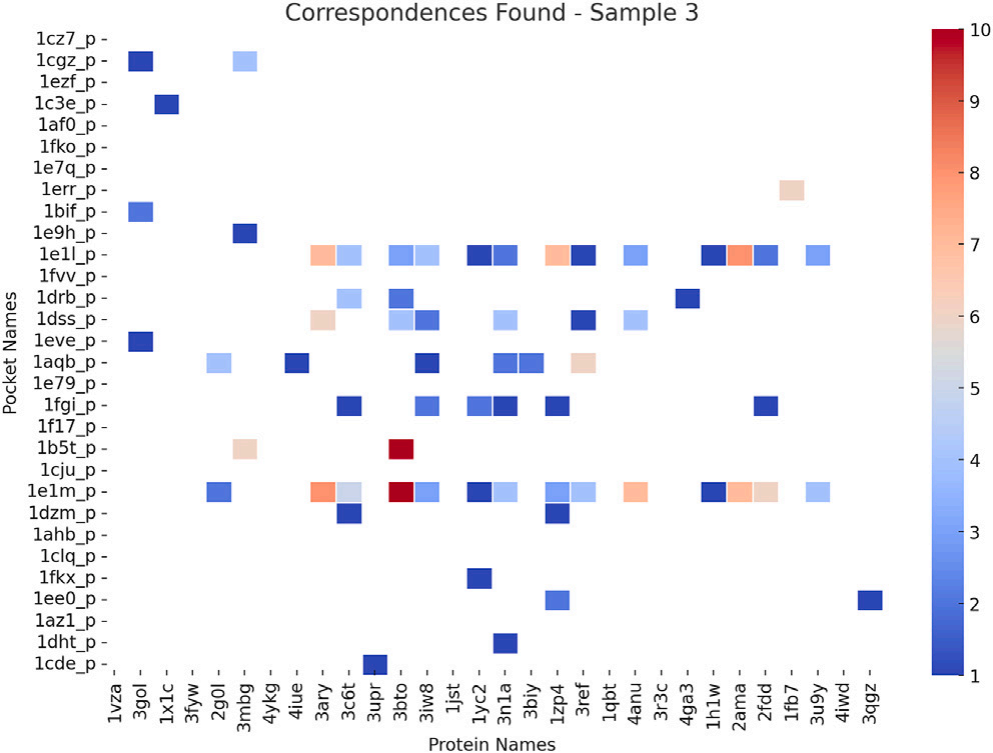
Heatmap showing a sample of similarity estimates between proteins and protein pockets in the scPDB database^25^

**Figure 16:**
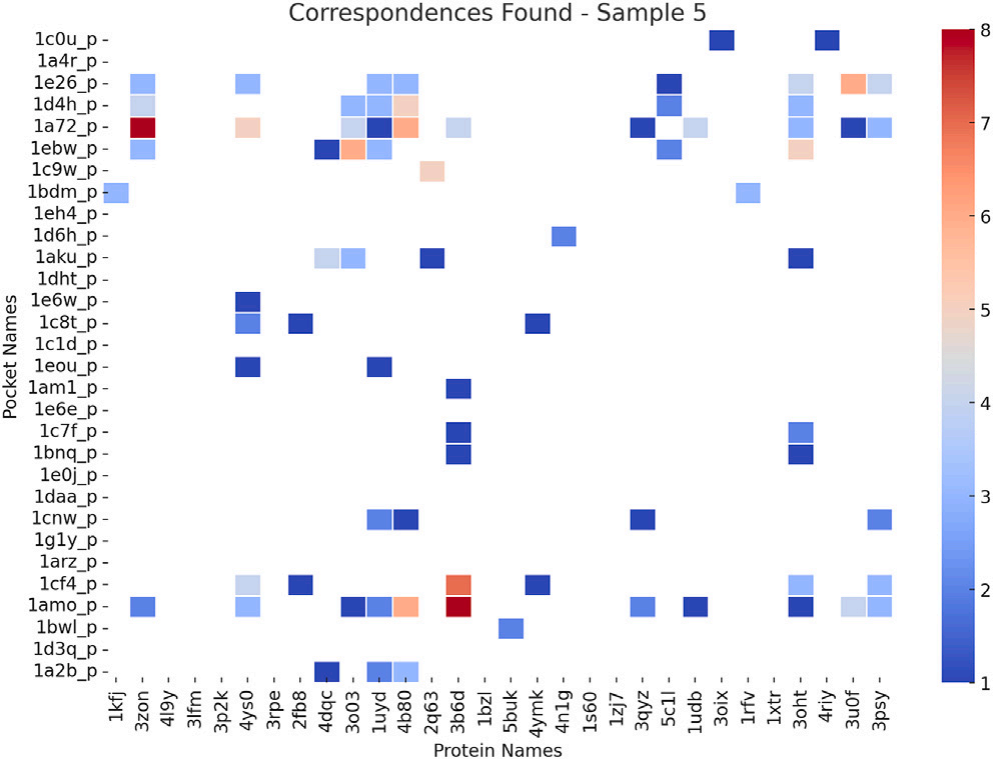
Heatmap showing a sample of similarity estimates between proteins and protein pockets in the scPDB database^25^

**Figure 17:**
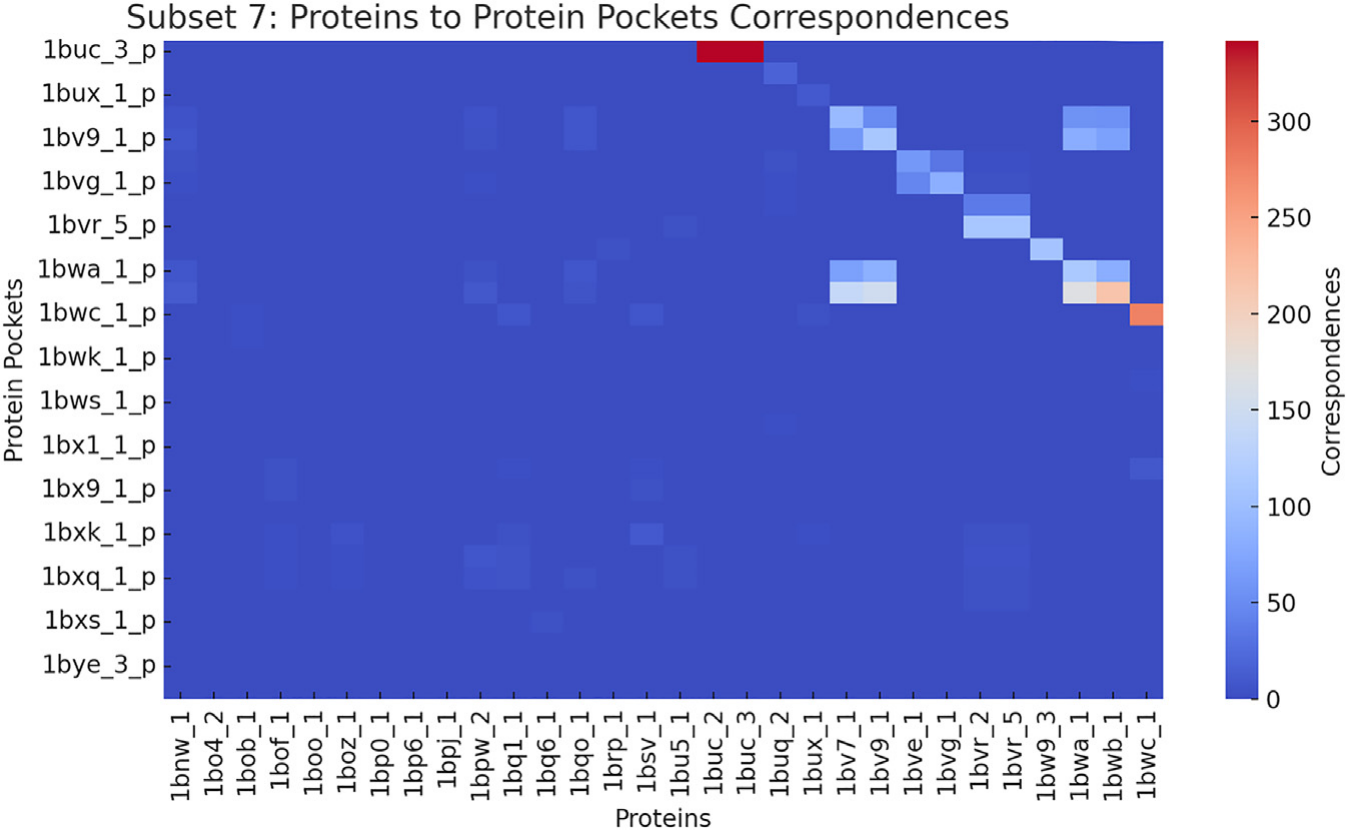
Heatmap showing a sample of similarity estimates between proteins and protein pockets in the BioLip database^26^^,^^27^^,^^28^

Protein-ligand binding is fundamental to structural bioinformatics and drug discovery. Understanding the spatial and functional similarities between different protein pockets provides crucial insights into their binding properties, potential cross-reactivity, and functional relationships. Figures 15, 16, and 17 present heatmaps illustrating similarity estimates between protein pockets and their corresponding proteins, based on a subset of experimental results from the scPDB and BioLip databases. The heatmaps use color intensity to represent similarity scores: deeper red indicates a higher degree of similarity, signifying strong structural resemblance, whereas lighter shades suggest lower similarity, reflecting structural or functional divergence.

From a bioinformatics perspective, high similarity scores often indicate evolutionary conservation, structural homology, or functional redundancy among protein pockets. This is particularly relevant for enzyme families, receptor binding sites, and allosteric modulation, where structurally similar pockets may engage with different ligands under varying physiological conditions. A high degree of pocket similarity also suggests potential ligand promiscuity, where multiple proteins share binding sites with similar physicochemical properties. This insight is essential in rational drug design, aiding in the identification of off-target interactions and cross-reactivity among different proteins.

For example, in Figure 15, protein 3bto exhibits strong similarity with pockets 1e1m_p and 1b5t_p, while in Figure 16, protein 3b6d shows strong similarity with pockets 1amo_p and 1cf4_p. Such similarity suggests the presence of conserved binding motifs, implying that these proteins may recognize and bind structurally similar ligands while potentially performing analogous biological functions. This resemblance can be leveraged for drug repurposing or predicting unknown binding interactions.

Conversely, protein 3b6d exhibits weak similarity with pocket 1a72_p and even weaker similarity with pockets 1am1_p, 1c7f_p, and 1bnq_p, indicating only partial structural compatibility. This suggests that while these proteins may share some overlapping ligand-binding potential, differences in pocket geometry, electrostatics, or residue composition likely affect ligand specificity and binding affinity.

In contrast, Figure 17 highlights a case where protein 1buc_3 exhibits strong similarity only with its own pocket, 1buc_3, and no resemblance to other pockets. This uniqueness suggests that 1buc_3 has a highly specialized function, possibly binding a distinct ligand not shared by other analyzed proteins. In drug discovery, such unique binding pockets may present challenges for ligand design due to their specialized structural features.

The estimation of binding pocket similarity has significant implications in structural bioinformatics and drug design. Proteins with strongly similar pockets may exhibit functional redundancy or convergence, interacting with similar molecules in biological pathways. This insight facilitates protein functional inference and pathway analysis. Additionally, structurally similar pockets can enable ligand repurposing, allowing the same inhibitors or drugs to effectively target multiple proteins, whereas unique pockets necessitate novel drug designs. From an evolutionary perspective, conserved pocket structures suggest functional preservation, while unique pockets imply divergent evolution and specialization.

Thus, analyzing pocket similarity provides valuable insights into protein function, ligand specificity, and drug discovery potential, reinforcing its role as a key approach in bioinformatics research.

### Real-world source exploration

To further demonstrate the practical applicability of our method, we conducted an additional experiment using real-world protein data. Drawing inspiration from previous studies,^29^^,^^30^^,^^31^^,^^32^^,^^33^ we applied our approach to a subset of H2B histone proteins to evaluate their binding pocket similarity. Given their essential role in chromatin organization and epigenetic regulation, histone proteins represent key targets in drug discovery. Identifying structurally similar binding pockets can provide valuable insights for the development of epigenetic inhibitors.

**Figure 18:**
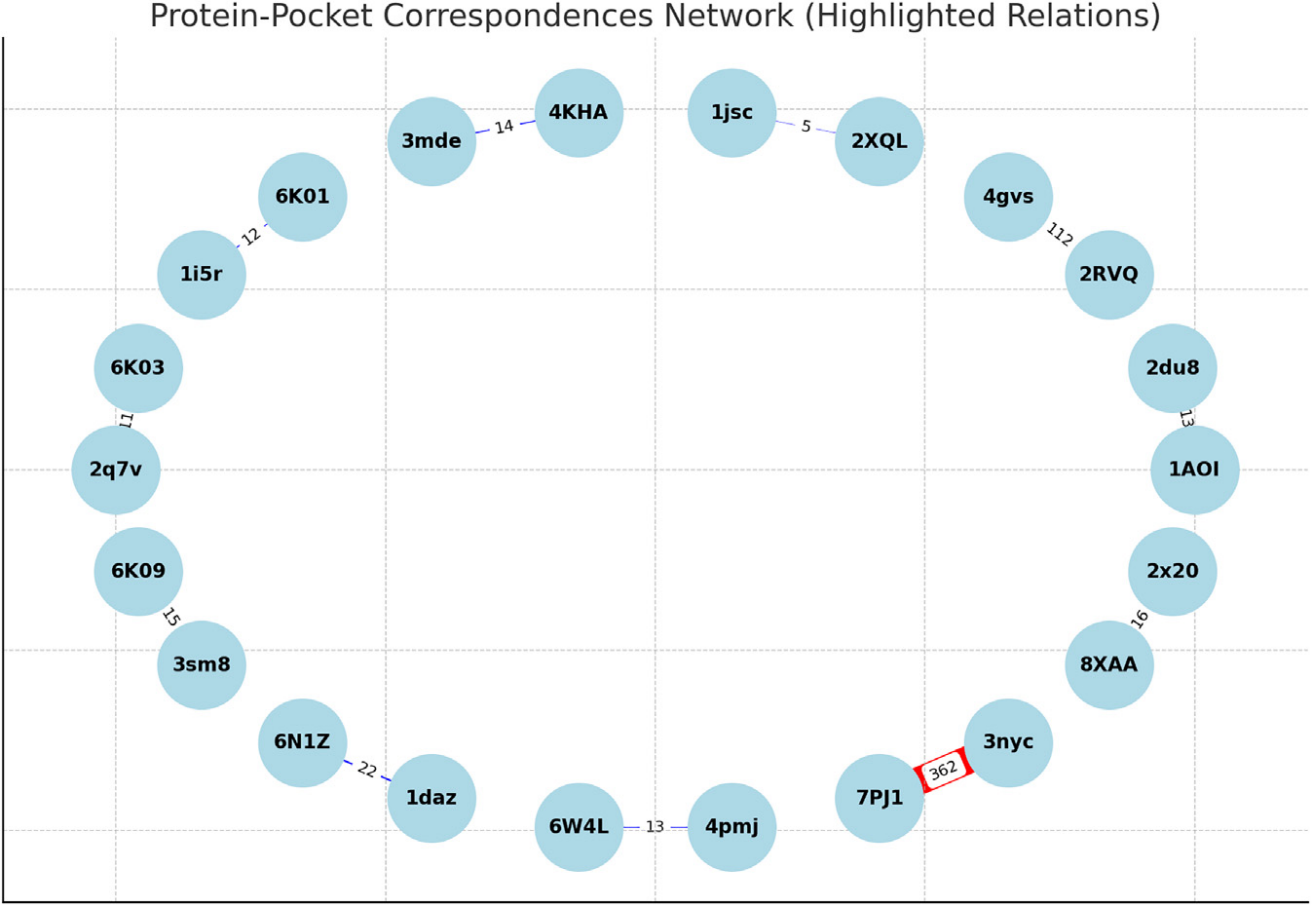
Similarity estimation results for a subset of H2B histone proteins

Figure 18 presents the similarity analysis results, categorizing matched binding pockets into strong and weak similarity groups based on computed correspondence scores. The analysis reveals that most histone proteins exhibit strong binding pocket similarity, with correspondence counts exceeding the threshold of 10. Notably, H2B histone protein 7PJ1 shows the highest number of correspondences (362), indicating a highly conserved or structurally similar binding pocket compared to its reference. In contrast, H2B histone protein 2XQL has the lowest number of correspondences (5), classifying it as a weak similarity case.

The identification of structurally similar binding pockets in histone proteins carries significant implications for drug discovery. Since histones play a fundamental role in chromatin remodeling and gene regulation, understanding the conservation of their binding sites can inform the design of targeted small molecules or epigenetic inhibitors. The presence of strong similarity across multiple histone variants suggests the potential for repurposing existing histone-binding compounds to target multiple histone subtypes.

Looking ahead, we aim to refine the similarity classification process by incorporating additional judgment criteria. Beyond geometric similarity, future assessments will consider binding capability, physicochemical compatibility, and ligand interaction profiles. The integration of molecular dynamics simulations and pharmacophore modeling will further enhance the predictive power of this approach. These advancements will contribute to a more comprehensive assessment of histone protein-ligand interactions, ultimately supporting structure-based drug design efforts in epigenetic therapeutics.

### Conclusion

The aim of this study was to develop an innovative computational approach for mining spatial geometric information within the 3D structures of proteins, enabling precise identification and characterization of pockets at the atomic level. We introduced a computer vision-based method named ProCV, which leverages 3D similarity grouping within 3D Hough space for effective pocket detection. ProCV was rigorously evaluated on benchmark datasets comprising diverse proteins and ligands across multiple experiments, demonstrating its capability to extract structural geometric information critical for protein-ligand binding site prediction. A notable strength of this method lies in its minimal hardware requirements and low algorithmic complexity, making it accessible for a wide range of computational environments. Beyond protein pocket detection, ProCV also facilitates similarity-based validation between different proteins and their corresponding binding pockets, enhancing the accuracy of comparative structural analyses.

Future research will focus on refining 3D feature extraction algorithms, alongside optimizing the weights and biases of fully connected layers in deep learning models for forward propagation. These improvements aim to enhance the prediction of structural features based on four-dimensional spatial and temporal data, thereby allowing adaptability to dynamic changes in the protein environment. Furthermore, we plan to explore novel cross-analysis techniques to monitor the evolution of protein pockets across various temporal and spatial scales. The ultimate objective is to develop methods for automatic, scalable, and high-throughput visualization of pocket features, providing a robust framework for advancing protein molecular docking predictions and facilitating drug discovery processes.

## Discussion

Traditional approaches in bioinformatics have primarily focused on characterizing and predicting the one-dimensional structural features of proteins. These methods include secondary structure prediction based on main-chain hydrogen bonding patterns, local structure modeling of tetrapeptides using pseudo-bond representations, dihedral angle-based residue conformation predictions, and assessments of the accessible surface area of residues in solvent environments. Such techniques serve as foundational features for characterizing target proteins, which are then used to inform higher-order predictions, including tertiary structure determination. The accuracy of these initial predictions is crucial as they directly impact the precision of the resulting three-dimensional (3D) structural models.

With the advent of advanced computational models, particularly AlphaFold, the scope of structural prediction has expanded significantly. The AlphaFold Protein Structure Database now offers near-comprehensive coverage of known protein structures, effectively integrating predicted models with existing structural repositories. This integration not only enhances the accessibility of structural data but also facilitates the evaluation of protein-ligand interactions, making it a valuable resource for molecular docking studies. As these databases continue to evolve, they provide an essential foundation for exploring the structural basis of protein functions and interactions.

Future research is expected to delve deeper into the 3D conformations of protein complexes, including the analysis of interatomic distances, angles, and interaction networks within these assemblies. This shift toward a more detailed understanding of protein-protein and protein-ligand interactions will offer critical insights into the molecular mechanisms that govern complex formation. Structure-based predictive models will play a key role in refining the specificity of binding site conformations and the coordination geometry of interacting residues. Such advancements are pivotal for elucidating the biological activities of protein complexes, thereby holding great promise for applications in drug discovery and precision medicine.

### Limitations of the study

ProCV significantly improves protein pocket recognition using 3D similarity grouping and geometric consistency techniques, but it has several limitations. Its reliance on structural databases (PDB, scPDB, and BioLip) makes performance dependent on data quality, with structural inconsistencies from experimental techniques affecting pocket alignment. While optimized with KD-tree indexing and the 3D Hough transform, scalability challenges remain for large complexes or dynamic ensembles, which could be mitigated by adaptive sampling or parallelization. The method’s focus on static structures limits its applicability to flexible binding sites, suggesting potential enhancements through molecular dynamics or flexible docking. Lastly, although ProCV performs well in benchmarks, further validation across diverse ligand classes and refined similarity thresholds, possibly incorporating machine learning, could enhance its predictive accuracy and generalizability.

## Resource availability

### Lead contact

For further information and requests for resources or reagents, please contact the Lead contact, Zhenhao Wang (wangzhenhao@qut.edu.cn).

### Materials availability

This study did not generate any new unique reagents.

### Data and code availability

• The scripts used in this study are specifically tailored to the generated data and are available from the corresponding author upon request.
• The original code and experimental results have been deposited on Zenodo (https://doi.org/10.5281/zenodo.14803394).
• Any additional information required to reanalyze the data reported in this paper is available from the lead contact upon request.

## Acknowledgments

This work was supported in part by the 10.13039/501100007129Natural Science Foundation of Shandong Province under Grant ZR2021MF101. This research was funded by the 10.13039/501100004543China Scholarship Council under admission number [2022]25 and student number 202200810001.

## Author contributions

Z.W.: 3D modeling methodology, program development, writing – original draft, conceptualization, supervision, project administration, writing – review and editing, funding acquisition, and validation. T.N.: Writing – review and editing.

## Declaration of interests

The authors declare that they have no known competing financial interests or personal relationships that could have influenced the work reported in this paper.

## STAR★Methods

### Key resources table

**Table:** 

REAGENT or RESOURCE	SOURCE	IDENTIFIER
**Deposited data**		

Protein DataBank (PDB)	RCSB PDB^22^^,^^23^^,^^24^	rcsb.org
Druggable Binding Sites Database	scPDB^25^	bioinfo-pharma.u-strasbg.fr/scPDB
Ligand-Protein Binding Database	BioLip^26^^,^^27^^,^^28^	seq2fun.dcmb.med.umich.edu/BioLiP

**Software and algorithms**		

Point Cloud Library	PCL	pointclouds.org
ProCV: A 3D Similarity Grouping Method for Enhanced Protein Pocket Recognition and Ligand Interaction Analysis	Zenodo	https://doi.org/10.5281/zenodo.14803394

### Experimental model and study participant details

This study focuses on computational approaches for protein pocket recognition and does not involve biological experimental models or human participants.

### Method details

#### Protein pocket recognition methodology

(1) The ProCV framework, a 3D similarity grouping method, is designed to enhance protein pocket recognition by employing advanced spatial recognition techniques. ProCV utilizes a Hough space transformation, which aids in the precise localization of protein pockets through 3D similarity estimation. This method improves both the speed and accuracy of protein-ligand binding site identification, overcoming the limitations of traditional sequence-based approaches.

#### Datasets used

(1) Protein DataBank (PDB)^22^^,^^23^^,^^24^: This database serves as the primary repository for 3D structural data of biological macromolecules.
(2) scPDB^25^: Focuses on identifying druggable binding sites across protein structures, with data from over 16,000 binding sites in more than 9,000 proteins used in the experiment.
(3) BioLip^26^^,^^27^^,^^28^: Contains ligand-protein binding interactions, providing essential data for functional predictions and ligand docking studies. Used in the experiment with data from over 38,000 complexes.

#### Computational techniques

(1) Uniform spatial sampling: Keypoints are extracted from both protein and ligand structures using uniform spatial sampling techniques. This ensures consistent representation of the 3D structure by dividing the space into a grid and extracting points from high-density regions. The centroid grid method is used to determine the center of each cluster, enhancing the extraction of relevant features for similarity analysis.
(2) KD-tree indexing: A KD-tree data structure is employed to efficiently handle the spatial distribution of protein and ligand data, facilitating rapid similarity matching through nearest neighbor searches. This approach significantly speeds up the identification process by organizing the point cloud data in a hierarchical manner.
(3) 3D Hough transform: The 3D Hough transform is applied for geometric consistency analysis between protein pockets. This technique identifies the structural features that allow the alignment of protein and ligand pocket geometries. It improves the accuracy of pocket matching by using rotational matrices and translation vectors to align the structures in a 3D space.

#### Software and tools

(1) Computational libraries: Implemented using C++ with the Point Cloud Library (PCL) for processing proteins and protein pocket data (transformed from PDB to PCD format). The experiment in this article uses version pcl_1.13.1 of the PCL library.

### Quantification and statistical analysis

(1) Benchmarking with existing methods: Efficiency (processing times in milliseconds) was compared with established computational approaches (Figure 5).
(2) Evaluation metrics: Statistical analysis was performed using POS (Figure 6), F1-score (Figure 7), MCC (Figure 8), and RMSE (Figure 9).
(3) Noise robustness analysis: Assessed how similarity grouping is affected by structural perturbations (Figure 10).

### Additional resources

(1) Data availability: The original code and experimental results have been deposited on Zenodo (https://doi.org/10.5281/zenodo.14803394).
(2) Publicly available Structural Datasets: PDB, scPDB, and BioLip were used for analysis.
(3) Lead contact: For further inquiries, please contact Zhenhao Wang (wangzhenhao@qut.edu.cn).

## References

[1] Tupler R., Perini G., Green M.R. (2001). Expressing the human genome. Nature.

[2] Brady G.P., Stouten P.F. (2000). Fast prediction and visualization of protein binding pockets with pass. J. Comput. Aided Mol. Des..

[3] Hendlich M., Rippmann F., Barnickel G. (1997). Ligsite: automatic and efficient detection of potential small molecule-binding sites in proteins. J. Mol. Graph. Model..

[4] Laurie A.T.R., Jackson R.M. (2005). Q-sitefinder: an energy-based method for the prediction of protein–ligand binding sites. Bioinformatics.

[5] Meller A., Ward M.D., Borowsky J.H., Lotthammer J.M., Kshirsagar M., Oviedo F., Lavista Ferres J., Bowman G. (2023). Predicting the locations of cryptic pockets from single protein structures using the pocketminer graph neural network. Biophys. J..

[6] Zhang Z., Shen W.X., Liu Q., Zitnik M. (2024). Efficient generation of protein pockets with pocketgen. Nat. Mach. Intell..

[7] Comajuncosa-Creus A., Jorba G., Barril X., Aloy P. (2024). Comprehensive detection and characterization of human druggable pockets through novel binding site descriptors. bioRxiv.

[8] Hetényi C., Spoel D.v.d. (2011). Toward prediction of functional protein pockets using blind docking and pocket search algorithms. Protein Sci..

[9] Jackson R.M. (2002). Q-fit: a probabilistic method for docking molecular fragments by sampling low energy conformational space. J. Comput. Aided Mol. Des..

[10] Hernandez M., Ghersi D., Sanchez R. (2009). Sitehound-web: a server for ligand binding site identification in protein structures. Nucleic Acids Res..

[11] Jumper J., Evans R., Pritzel A., Green T., Figurnov M., Ronneberger O., Tunyasuvunakool K., Bates R., Žídek A., Potapenko A. (2021). Highly accurate protein structure prediction with alphafold. nature.

[12] Abramson J., Adler J., Dunger J., Evans R., Green T., Pritzel A., Ronneberger O., Willmore L., Ballard A.J., Bambrick J. (2024). Accurate structure prediction of biomolecular interactions with alphafold 3. Nature.

[13] Terwilliger T.C., Liebschner D., Croll T.I., Williams C.J., McCoy A.J., Poon B.K., Afonine P.V., Oeffner R.D., Richardson J.S., Read R.J., Adams P.D. (2024). Alphafold predictions are valuable hypotheses and accelerate but do not replace experimental structure determination. Nat. Methods.

[14] Varadi M., Bertoni D., Magana P., Paramval U., Pidruchna I., Radhakrishnan M., Tsenkov M., Nair S., Mirdita M., Yeo J. (2024). Alphafold protein structure database in 2024: providing structure coverage for over 214 million protein sequences. Nucleic Acids Res..

[15] Durairaj J., de Ridder D., van Dijk A.D.J. (2023). Beyond sequence: Structure-based machine learning. Comput. Struct. Biotechnol. J..

[16] Eguida M., Rognan D. (2020). A computer vision approach to align and compare protein cavities: application to fragment-based drug design. J. Med. Chem..

[17] Eguida M., Rognan D. (2022). Estimating the similarity between protein pockets. Int. J. Mol. Sci..

[18] Eguida M., Bret G., Sindt F., Li F., Chau I., Ackloo S., Arrowsmith C., Bolotokova A., Ghiabi P., Gibson E. (2024). Subpocket similarity-based hit identification for challenging targets: Application to the wdr domain of lrrk2. J. Chem. Inf. Model..

[19] Wang Z., Zhao Y., Wang S. (2019). H∞ optimal control-based robust pose estimation in light field three-dimensional display. IEEE Access.

[20] Wang Z., Zhao Y., Wang S. (2018). A multi-object oriented iterative closest point algorithm in augmented reality. Advances in Display Technologies VIII.

[21] Krapp L.F., Abriata L.A., Cortés Rodriguez F., Dal Peraro M. (2023). Pesto: parameter-free geometric deep learning for accurate prediction of protein binding interfaces. Nat. Commun..

[22] Murumkar P.R., Sharma M.K., Gupta P., Patel N.M., Yadav M.R. (2023). Selection of suitable protein structure from protein data bank: An important step in structure-based drug design studies. Mini Rev. Med. Chem..

[23] Bank P.D. (1971). Protein data bank. Nature. New. Biol..

[24] Yan Y., Tao H., He J., Huang S.Y. (2020). The hdock server for integrated protein–protein docking. Nat. Protoc..

[25] Desaphy J., Bret G., Rognan D., Kellenberger E. (2015). sc-pdb: a 3d-database of ligandable binding sites–10 years on. Nucleic Acids Res..

[26] Carpenter K.A., Altman R.B. (2024). Databases of ligand-binding pockets and protein-ligand interactions. Comput. Struct. Biotechnol. J..

[27] Zhang C., Zhang X., Freddolino L., Zhang Y. (2024). Biolip2: an updated structure database for biologically relevant ligand–protein interactions. Nucleic Acids Res..

[28] Yang J., Roy A., Zhang Y. (2013). Biolip: a semi-manually curated database for biologically relevant ligand–protein interactions. Nucleic Acids Res..

[29] Chakraborty S., Mahamid J., Baumeister W. (2020). Cryoelectron tomography reveals nanoscale organization of the cytoskeleton and its relation to microtubule curvature inside cells. Structure.

[30] Baumeister W. (2022). Cryo-electron tomography: A long journey to the inner space of cells. Cell.

[31] Bose M., Lampe M., Mahamid J., Ephrussi A. (2022). Liquid-to-solid phase transition of oskar ribonucleoprotein granules is essential for their function in drosophila embryonic development. Cell.

[32] Zhang X., Sridharan S., Zagoriy I., Eugster Oegema C., Ching C., Pflaesterer T., Fung H.K.H., Becher I., Poser I., Müller C.W. (2023). Molecular mechanisms of stress-induced reactivation in mumps virus condensates. Cell.

[33] Wang S., Chakraborty S., Fu Y., Lee M.P., Liu J., Waldhaus J. (2024). 3D reconstruction of the mouse cochlea from scrna-seq data suggests morphogen-based principles in apex-to-base specification. Dev. Cell.

[34] Ou W., Zheng M., Zheng H. (2024). Mdu-sampling: Multi-domain uniform sampling method for large-scale outdoor lidar point cloud registration. Electron. Lett..

[35] Zheng X., Huang X., Mei G., Hou Y., Lyu Z., Dai B., Ouyang W., Gong Y. (2024). Proceedings of the IEEE/CVF Conference on Computer Vision and Pattern Recognition.

[36] Wang Z., Xu R., Nie T., Xu D. (2023). Semi-supervised active learning hypothesis verification for improved geometric expression in three-dimensional object recognition. Eng. Appl. Artif. Intell..

[37] Bentley J.L. (1979). Multidimensional binary search trees in database applications. IEEE Trans. Software Eng..

[38] Wang Z., Zhao Y., Wang S. (2019). Approach for improving efficiency of three-dimensional object recognition in light-field display. Opt. Eng..

[39] Hamid N.A. (2024). Proceedings of the 39th ACM/SIGAPP Symposium on Applied Computing.

[40] Şenol A. (2024). Impkmeans: An improved version of the k-means algorithm, by determining optimum initial centroids, based on multivariate kernel density estimation and kd-tree. Acta Polytechnica Hungarica.

[41] Romanengo C., Falcidieno B., Biasotti S. (2024). Discretisation of the hough parameter space for fitting and recognising geometric primitives in 3d point clouds. Math. Comput. Simulat..

[42] Wu L., Li X., Zhong K., Li Z., Wang C., Shi Y. (2022). Hccg: Efficient high compatibility correspondence grouping for 3d object recognition and 6d pose estimation in cluttered scenes. Measurement.

[43] Azimi S., Gandhi T.K. (2021). 3-d maximum likelihood estimation sample consensus for correspondence grouping in 3-d plant point cloud. IEEE Sens. Lett..

[44] Hekkelman M.L., de Vries I., Joosten R.P., Perrakis A. (2023). Alphafill: enriching alphafold models with ligands and cofactors. Nat. Methods.

[45] Jiménez J., Doerr S., Martínez-Rosell G., Rose A.S., De Fabritiis G. (2017). Deepsite: protein-binding site predictor using 3d-convolutional neural networks. Bioinformatics.

[46] Le Guilloux V., Schmidtke P., Tuffery P. (2009). Fpocket: an open source platform for ligand pocket detection. BMC. Bioinformatics..

[47] Weisel M., Proschak E., Schneider G. (2007). Pocketpicker: analysis of ligand binding-sites with shape descriptors. Chem. Cent. J..

[48] Huang L., Wang C., Yun J., Tao B., Qi J., Liu Y., Ma H., Yu H. (2023). Object pose estimation based on stereo vision with improved k-d tree icp algorithm. Concurrency Comput. Pract. Ex..

